# Dysfunctional serotonergic neuron-astrocyte signaling in depressive-like states

**DOI:** 10.1038/s41380-023-02269-8

**Published:** 2023-09-29

**Authors:** Candela González-Arias, Andrea Sánchez-Ruiz, Julio Esparza, Cristina Sánchez-Puelles, Lucia Arancibia, Jorge Ramírez-Franco, Davide Gobbo, Frank Kirchhoff, Gertrudis Perea

**Affiliations:** 1 Cajal Institute, CSIC, 28002 Madrid, Spain; 2 PhD Program in Neuroscience, Autonoma de Madrid University-Cajal Institute, Madrid, 28029 Spain; 3 Tetraneuron, 46005 Valencia, Spain; 4 Institut de Neurosciences de la Timone, Aix-Marseille Université (AMU) & CNRS, UMR7289, 13005 Marseille, France; 5 Molecular Physiology, Center for Integrative Physiology and Molecular Medicine, University of Saarland, 66421 Homburg, Germany

**Keywords:** Neuroscience, Biological techniques

## Abstract

Astrocytes play crucial roles in brain homeostasis and are regulatory elements of neuronal and synaptic physiology. Astrocytic alterations have been found in Major Depressive Disorder (MDD) patients; however, the consequences of astrocyte Ca^2+^ signaling in MDD are poorly understood. Here, we found that corticosterone-treated juvenile mice (Cort-mice) showed altered astrocytic Ca^2+^ dynamics in mPFC both in resting conditions and during social interactions, in line with altered mice behavior. Additionally, Cort-mice displayed reduced serotonin (5-HT)-mediated Ca^2+^ signaling in mPFC astrocytes, and aberrant 5-HT-driven synaptic plasticity in layer 2/3 mPFC neurons. Downregulation of astrocyte Ca^2+^ signaling in naïve animals mimicked the synaptic deficits found in Cort-mice. Remarkably, boosting astrocyte Ca^2+^ signaling with Gq-DREADDS restored to the control levels mood and cognitive abilities in Cort-mice. This study highlights the important role of astrocyte Ca^2+^ signaling for homeostatic control of brain circuits and behavior, but also reveals its potential therapeutic value for depressive-like states.

## Introduction

Astrocytes have emerged as active modulatory cells in synaptic transmission and plasticity [1, 2]. By sensing synaptic activity through Ca^2+^-dependent [3] and independent [4] mechanisms, astrocytes respond to different neurotransmitters triggering a wide range of cellular responses, including the release of active substances, so-called gliotransmitters [5]. These gliotransmitters have been found to regulate neuronal excitability and synaptic physiology [6], impacting brain activity and animal behavior [7]. Recent evidence has shown the crucial role that astrocytic activity plays in complex behaviors, from motor [8] and sensory processing [9], to cognitive [10] and emotional responses [11]. Such broad range of astrocyte functions indicates their ability to adjust their physiology to continuously meet the varying demands of neural activity [12]. Indeed, astrocytes are highly sensitive to brain stressors, undergoing functional and structural changes, which emphasizes their role in neurological and psychiatric diseases [13].

Stress stimulates the hypothalamic-pituitary-adrenal (HPA) axis and the secretion of stress hormones, that is, cortisol in humans [14] and corticosterone in murine animals [15], which act on glucocorticoid receptors. Astrocytes are enriched in glucocorticoid receptors [16] making them an ideal target for corticosterone actions. In fact, acute and chronic stress exposure induces significant alterations in astrocyte physiology, including alterations in connexins expression, glutamate uptake, glucose metabolism or purinergic signaling among others [16–19]. Chronic stress is one of the main factors related with Major Depressive Disorder (MDD) [15, 20–22], a severe mental illness leading to cognitive impairments and psychosocial alterations. In addition to the reported decreased social interaction and impaired emotional information processing [23], MDD is associated with alterations in attention, executive functions and learning and memory processes [24]. In particular, stressful events during childhood and adolescence have critical impact in brain maturation, and are related to later social and emotional maladjusted behaviors, including depressive-disorders [25, 26]. In animal research, different chronic stress protocols are commonly used to study anxiety and depressive phenotypes [27, 28], including a corticosterone treatment [29–31], allowing to evaluate the therapeutical value of antidepressants [32, 33]. Currently, the main drugs to treat depression target the serotonergic system, increasing serotonin (5-HT) availability in the synaptic cleft as a common effect [34], supporting the monoaminergic hypothesis of depression [35]. Although other therapeutic strategies have been developed as antidepressant drugs targeting the glutamatergic system, AMPA and NMDA receptors [36, 37]; GABAergic system, GABAa receptors [38]; glucocorticoids receptors [39]; 5-HT receptors [40] and dopamine receptors D2 [41]. The currently available treatments are only effective in half of MDD patients, revealing the complex heterogeneous nature of the disorder, which possibly involves multiple etiologies [42]. Therefore, fully understanding the multisided mechanism responsible for the development of depression is crucial to develop new therapeutic strategies.

To add complexity, postmortem analyses of MDD patients have shown reductions of astrocytic densities in multiple brain areas [43, 44]. Moreover, S100β, a calcium binding protein mainly found in astrocytes, was elevated in the serum of MDD patients [45], indicating that astrocytes may be relevant actors in the pathogenesis of MDD [46]. In this line, studies performed in animal models showed that experimental manipulations to reduce astrocyte densities in medial prefrontal cortex (mPFC) induced depressive-like phenotypes [47]. Furthermore, the pharmacological blockade of connexins, glutamate uptake, or K^+^ homeostasis alterations induced depressive-like behaviors [18, 48–50]. Reduced levels of ATP in the extracellular space from neuronal or astrocyte sources have also been related to the pathophysiology of MDD [51]. Altogether, these evidences indicate that astrocytes contribute to MDD through different molecular pathways. Nevertheless, little is known about how this pathological brain state impacts astrocyte Ca^2+^ signaling, a key intracellular regulator of astrocyte physiology, both in resting and in response to neuronal demands [17].

Here, we investigated the astrocyte Ca^2+^ dynamics and 5-HT-driven astrocyte-neuron signaling in a chronic corticosterone mouse model of stress, Cort-mice, that recapitulates depressive-like behaviors [29–31, 33]. We have focused on mPFC, a critical hub for executive function and emotion regulation, which is a target for stress hormones, and it has been found implicated in many stress-influenced psychological disorders, including MDD [52, 53]. In juvenile-treated mice, we found abnormal in vivo astrocyte Ca^2+^ signaling in mPFC in Cort-mice, which was largely impaired during social interactions. Ex vivo recordings in mPFC slices confirmed the dysfunctional astrocytic signaling in depressive-like states, with enhanced oscillatory activity but reduced amplitude of Ca^2+^ events in Cort-mice, and diminished 5-HT-engaged astrocytic Ca^2+^ activity. Additionally, Cort-mice showed an altered 5-HT-driven synaptic plasticity in layer 2/3 mPFC excitatory neurons, that was replicated by downregulating astrocyte Ca^2+^ signaling in control mice. Remarkably, the cognitive and mood abilities altered in Cort-mice were restored to normal by selective chemogenetic (Gq-DREADDS) manipulation of astrocyte Ca^2+^ signaling in vivo. By contrast, significant behavioral impairments were found in naïve mice after chemogenetic astrocyte activation, revealing that the subtle control of astrocyte Ca^2+^ signaling is crucial for the proper function of mPFC in health and disease. Altogether, this study shows the prominent role of astrocytes for the serotonergic system and highlights the potential of astrocytic intracellular Ca^2+^ modulation as a therapeutical target for depressive-like states.

## Methods

### Experimental subjects

All the procedures for handling and sacrificing animals followed the European Commission guidelines for the welfare of experimental animals (2010/63/EU) and were approved by the local Bioethics Committee (2013/53/RD). Animals were housed in standard laboratory cages with *ad libitum* access to food and water, under a 12-hour dark–light cycle in temperature-controlled rooms. Male C57BL/6 wild-type mice, *Ip3r2*^*−/−*^ (RRID: MGI:3640970), and *Aldh1l1*-EGFP mice (RRID: MMRRC_011015-UCD) were used in the present study from 1-3 months old. Mice studied for longitudinal analysis of corticosterone treatment effects (Extended data Fig. 1a, b) were 5 months old. C57BL/6 mice were purchased from Jackson Laboratory. *Ip3r2*^*−/−*^ mice were generated by crossing germline-heterozygous-null mutant *Ip3r2*^+/−^ mice [54].

### Corticosterone treatment

Corticosterone (Cort, Cat# C2505; Sigma-Aldrich) was dissolved in commercial mineral water [55]. Decreasing Cort concentrations were presented to male C57BL/6 wild-type mice for 28 days: 30 μg/ml during 15 days (resulting in a dose of approximately 6.6 mg/kg/day), followed by 15 μg/ml (2.7 mg/kg/day) during 3 days, and 7.5 μg/ml (1.1 mg/kg/day) during 10 days; for a gradual recovery of endogenous corticosterone plasma levels [33, 55]. Cort solution was available *ad libitum* in drinking water (dark bottles) and was renewed every 72 h. To verify Cort consumption, bottles´ weight was controlled each time the solution was renewed. Control mice (naïve) followed the same experimental approach without Cort in their bottles. At the end of the treatment, mice from both groups were subjected to behavioral tests. Cort treatment started at P30. Forced swimming test (FST) was routinely evaluated to highlight the level of animal despair in order to guarantee the existence of a reliable mouse model of depression [33, 55]. In a subset of mice, long-term effects of Cort treatment were analyzed after 3 months (Extended Data Fig. 1a). After two weeks of treatment, naïve and Cort-mice were injected with viral vectors. Mice were randomly distributed in naïve and Cort-group before the treatment, and animal weight distribution was equally represented in both groups.

### Corticosterone measurements

Glucocorticoids deposited in hair have been identified as a biomarker-based stress assessment (cf. [56]). Corticosterone (Cort) was measured in hair and serum samples. Both samples were collected before the start of the treatment and 1- and 90-days post Cort treatment. Hair samples were obtained in anesthetized mice by shaving dorsal midline using an electric razor and stored at 4 °C. Hair samples were washed three times with isopropanol and gently mix followed by decanting excess isopropanol, and allowed to dry for 48 h. Dried hair samples were transferred to 2 mL polypropylene tubes containing 2 steel ball to ground to a fine powder at 30 Hz for 30 min. In total, 50 mg of ground powdered hair samples were placed in centrifuge tube containing 1 mL of methanol and kept on rotation overnight at room temperature (RT). Samples were centrifuged at 4000 rpm for 10 min, at 25 °C and 0.5 mL of the steroid-containing methanol supernatant was transferred to a new polypropylene tube and evaporated during 1 h at 30 °C. The dried extracts were reconstituted in 100 µL of Assay buffer provided in the corticosterone enzyme immunoassay kit.

Blood samples were collected by cardiocentesis after euthanasia by CO_2_ overdose between 09:00-10:00 am. Blood was centrifuged at 9000 × *g* for 10 min at 4 °C, and serum was separated and stored at –80 °C until analysis.

Corticosterone levels in hair and serum samples were quantified using a commercially available enzyme immunoassay kit (DetectX® Corticosterone Enzyme Immunoassay Kit K014-H5 Arbor Assays). Samples were analysed in duplicate according to the manufacturer’s instructions.

### Surgeries and viral injections

Mice (1–2 months) were anesthetized via isoflurane (5% for induction, 2% for maintenance) in oxygen and place in a custom adapted stereotaxic frame. Depth of anesthesia was determined by testing toe-pinch reflexes. Body temperature was continuously monitored and maintained at 37 °C. Buprenorphine (0.1 mg/kg; Buprenex, 0.1 mg/ml) was subcutaneously injected before surgeries. The hair of the scalp was shaved and cleaned with 70% ethanol. Once bregma and lambda were exposed, target coordinates were taken from Paxinos atlas [57]. Medial prefrontal cortex (mPFC) coordinates: 1.78 mm anterior, ±0.3 mm lateral from bregma, and from 2.5 to 0.8 dorso-ventral; Dorsal raphe nucleus (DRN) coordinates: posterior coordinate was scaled using bregma-lambda distance x 0.98 for every mouse [58], 1.1 mm lateral from lambda, and from 3.3 to 2.8 dorso-ventral, 20° angle. A craniotomy was made at the injection site using a small burr (Ref. 19007-05, Fine Science Tools), powered by a surgical drill (NSK V-Max Volvere Lab System). Saline solution (0.9%) was applied to keep the skull cold and to maintain hydration. Intracranial injections were made using a borosilicate micropipette (World Precision Instuments) at 50 nl/min infusion rate. The following viral vectors were used: AAV5-gfaABC1D-cyto-GCaMP6f (Addgene 52925; viral titer 1.3 × 10^13^ GC/ml), AAV5-GFAP-hM3Dq-mCherry (Addgene 50478; viral titer 2 × 10^13^ GC/ml), AAV8-GFAP104-mCherry (UNC Vector Core; viral titer 2.7 × 10^12^ GC/ml), AAV9-hSyn-ChrimsonR-tdTom (UNC Vector Core; viral titer 4.1 × 10^12^ GC/ml), AAV5-GFAP-mcherry-cre (UNC Vector Core; viral titer 4.3 × 10^12^ GC/ml), AAV5-CAG-flex-iSeroSnFR (Addgene 128486; viral titer 5 x + 10^12^ GC/ml, Unitat de Vectors Virals, UAB), AAV5-GFAP-eGFP-WPRE-hGH (Addgene 105549; viral titer 1.3 × 10^13^ GC/ml), AAV9-CaMKIIa-eYFP (Penn Vector Core, viral titer 2.55 × 10^12^ GC/ml). After injection, the micropipette was held in place for 5 min prior to retraction to prevent leakage. The skin was sutured and the mice were monitored, kept on a heating pad while recovering and returned to the home cage. Buprenorphine (0.05 ml, 0.1 mg/ml), was given once daily for 48 h post-surgery. Experiments were performed 2–3 weeks post-injection. Viral injections were performed bilaterally, but for in vivo recordings involving cannula implantations only the right hemisphere was selected.

### Cortical slice preparation

Animals were sacrificed and their brains were quickly and carefully removed [59]. The brains were placed in an ice-cold artificial cerebrospinal fluid (aCSF) containing [in mM]: NaCl 124, KCl 2.69, KH_2_PO_4_ 1.25, MgSO_4_ 2, NaHCO_3_ 26, CaCl_2_ 2, and glucose 10, and was gassed with carbogen (95% O_2_/5% CO_2_, pH = 7.3). Slices 350 μm thick were obtained with a vibratome (Leica Vibratome VT1200S, Germany) and incubated (>1 h) at RT (22–24 °C) in aCSF continuously bubbled. Slices were then transferred to an immersion recording chamber superfused at 2 ml/min with gassed aCSF and visualized under an Olympus BX50WI microscope (Olympus Optical, Japan) coupled with a 40x water immersion lens and infrared-DIC optics.

To improve slice viability in adult mice (>2 months old), ice-cold (4 °C) NMDG-HEPES solution was perfused prior to brain extraction. Subsequently, slices were placed in the same NMDG-HEPES solution at 37 °C for 10 min. Afterwards, slices were kept in aCSF until use (>1 h). NMDG-HEPES contained the following [in mM]: NMDG 92, KCl 2.5, NaH_2_PO_4_ 1.2, NaHCO_3_ 30, HEPES 20, glucose 25, thiourea 2, Na-ascorbate 5, Na-pyruvate 3, CaCl_2_·2H_2_O 0.5, and MgSO_4_·7H_2_O 10 (95% O_2_/5% CO_2_, pH = 7.3) [60]. A modified Mg^2+^-free aCSF was used to monitor slow inward currents (SICs) in order to optimize NMDA receptor activation, which contained the following [in mM]: NaCl 124, KCl 2.69, KH_2_PO_4_ 1.25, NaHCO_3_ 26, glucose 10, CaCl_2_ 4 and glycine 0,01 (95% O_2_/5% CO_2_, pH = 7.3).

### Ex vivo calcium imaging and analysis

The genetically encoded calcium indicator (GECIs) AAV5-GFAP-cytoGCaMP6f was bilaterally injected in mPFC, and after 2–3 weeks astrocytes from cortical slices were analyzed. Astrocytes of mPFC layer 2/3 were imaged using a CCD camera (ORCA-235, Hamamatsu, Japan) attached to the microscope. Cells were illuminated for 100–200 ms at 490 nm using LED system (CoolLED pE-100), and images were acquired at 1 Hz during 2 min. The LED system and the camera were controlled and synchronized by NIS Elements software (Nikon, Japan). Spontaneous Ca^2+^ events were monitored during 2 min in presence of TTX; and Ca^2+^ evoked responses were analyzed by recording baseline activity for 30 s, followed by local application of 5-HT (1 mM; 10 s, 1 bar), ATP (1 mM; 10 s, 1 bar), and clozapine-N-oxide (CNO, 1 mM; 2 s, 1 bar) restricted to 60 s after stimuli. Local application of agonists was delivered by pressure pulses through a micropipette (Picospritzer II, Parker Hannifin, Mayfield Heights, OH, USA). To isolate the specific response of astrocytes to 5-HT, the following drug cocktail was included in aCSF: TTX 1 µM, picrotoxin 50 µM, AM251 2 µM, MRS 2179 10 µM, CGP 55845 5 µM, LY367385 100 µM. For ATP experiments, MRS 2179 was excluded from the cocktail. For CNO experiments, TTX was added to the aCSF. For the experiments combining Ca^2+^ imaging and optogenetic stimulation, baseline recordings were acquired for 60 s, and astrocyte-evoked responses were considered up to 60 s after DRN fibers stimulation.

Regions of interest (ROI) were manually selected using ImageJ software. All pixels within each ROI were averaged to obtain a single time course F[t] per ROI. Custom-written software in MATLAB (MATLAB R2020a; Mathworks, Natick, MA) was used for further processing (modified from Mederos et al., 2020). Artifacts in the fluorescence signal produced by mechanical movement were removed from the analysis. Then, signals were low-pass filtered with a Chebyshev II filter. Photobleaching was adjusted and the ΔF/F_0_ was calculated for each ROI. Events were considered when their ΔF/F_0_ > 2-3 times the noise variance and had at least > 3% of relative change (0.03). Frequency, amplitude, area under the curve, and duration were analyzed for each ROI. In a subset of experiments (Extended Data Fig. 2a, b and Extended Data Fig. 3e, f), mPFC slices were incubated with Fluo-4 AM (1 μl of 2 mM dye was dropped over the mPFC, attaining a final concentration of 2-10 μM) dissolved in 0.02% pluronic and 0.04% DMSO for 15-20 min at RT, and Ca^2+^ signal analysis was restricted to the cell soma (cf. [61]).

### In vivo calcium recordings and analysis

AAV5-GFAP-cyto-GCaMP6f was injected in the right hemisphere of mPFC, followed by implantation of 2 mm borosilicate fiber-optic cannulas (fiber core Ø of 400 µm; 0.66 NA; ref. MFC_400/430-0.66_2.0_MF1.25_FLT, Doric Lenses). Cannulas were secured to the skull using a base layer of adhesive dental cement (Meron, Voco). 2-3 weeks after the surgery, behavioral testing started to allow for viral expression and animal recovery.

Doric GCaMP Fiber Photometry System (FPS_1S_GCaMP, Doric Lenses) was used, with a 405 nm LED as the isosbestic point, and a 465 nm LED as the excitation dependent GCaMP fluorescence. Blue light was delivered to the brain at 20–50 µW. Signals were interleaved and collected at 100 Hz. Raw signals were demodulated and analyzed with custom-written software in MATLAB, with a cut-off frequency of 20 Hz and an attenuation of 20 dB, followed by a 1 s moving mean window. Isosbestic signals were fitted to Ca^2+^-dependent signals and subtracted to eliminate motion related artifacts [62, 63]. GCaMP6 fluorescence signals across animals were standardized as follows: ΔF=(F-F_0_)/F_0_, where F_0_ was computed by linearly interpolating between the local minima of the fluorescence signal across different time windows (window size: 45 s) to account for any remaining photobleaching. Ca^2+^ event was defined as a period in which fluorescence showed a local maximum >2 times the noise variance of the signal [64]. Events whose maximum value was below 0.01 (1% of relative change), or whose prominence were below 0.001 (0.1%) were excluded. Events were expanded towards the closest local minima (both before and after the peak), to designate the start and end of putative Ca^2+^ events. To account for multipeak events, Gaussians were fitted to each Ca^2+^ event to infer their real duration and area under the curve.

**Fig. 1:**
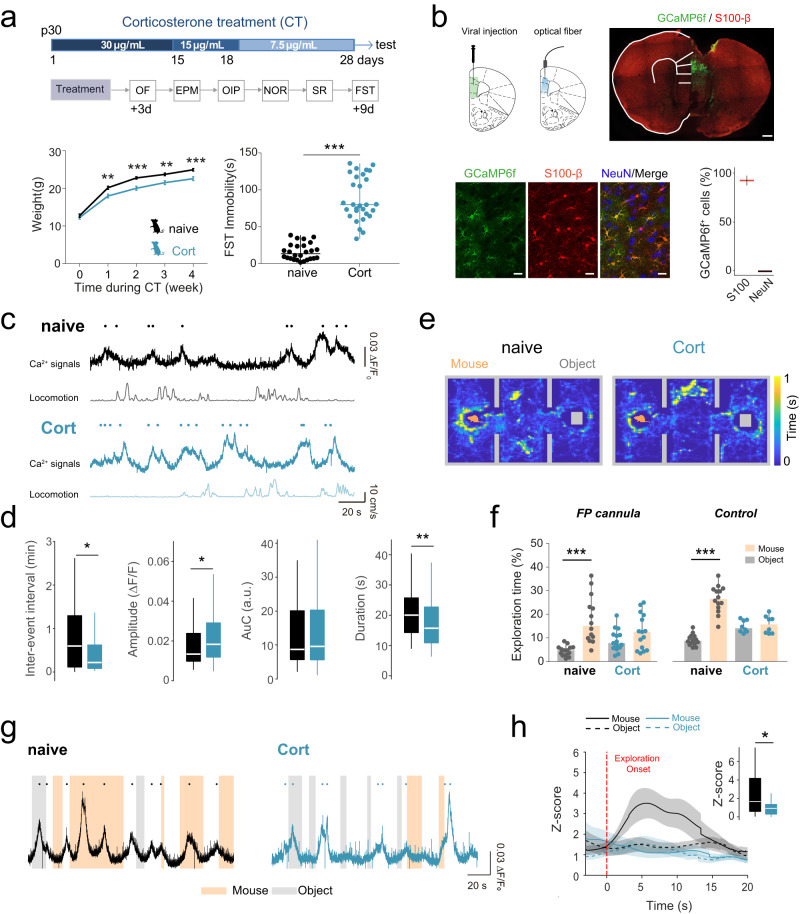
Altered astrocytic Ca^2+^ dynamics during social interactions in Cort-mice. **a** Top, Scheme of corticosterone (Cort) treatment protocol for 28 days followed by behavioral testing. Decreased doses of Cort were applied at different time points. Behavioral testing schedule. OF, EPM, OIP, NOR, SR and FST were conducted sequentially from 3 days (OF) to 9 days after treatment (FST). Bottom left, body weights for naïve (in black, as in the rest of the figure) and Cort-mice (in blue, as in the rest of the figure) were monitored during the entire Cort treatment. Naïve mice were exposed to water as control. Note the reduced gain of weight for Cort-mice (*n* = 26 for naïve; *n* = 29 for Cort). One Way ANOVA, Holm-Sidak method. *P* = 0.002, *P* < 0.001. Data are presented as mean ± s.e.m. Bottom right, cumulative duration of immobility during force swimming test (FST) in naïve (*n* = 26) and Cort mice (*n* = 29), showing an increased immobility rates compare to their naïve littermates. One Way ANOVA, Dunn’s test. *P* < 0.001. Data are shown as median ± range (min and max values). **b** Top, schematic representation of mPFC area targeted with AAV5-GFAP-GCaMP6f viral injection and optical fiber implantation in naïve and Cort-mice. Confocal image showing the selective viral expression (green) in astrocytes (S100β, red) in mPFC. Scale bar: 500 μm. Bottom left, representative confocal images showing GCaMP6f expression and astrocytic (S100β) and neuronal markers (NeuN) labeling. Scale bar: 20 μm. Bottom right, colocalization analysis of GCaMP6f positive cells and S100β and NeuN labeling (162 GCaMP6f positive cells from 3 slices, 3 mice; One Way ANOVA, Tukey test, *P* < 0,001). **c** Representative in vivo astrocytic Ca^2+^ activity traces from fiber photometry recordings and speed in naïve (top) and Cort-mice (bottom) during open field explorations. Dots denote the peak of astrocyte Ca^2+^ events. **d** Box and whisker (BW) plots analysis of Ca^2+^ dynamics in both groups (naïve= 7, Cort= 6). One Way ANOVA, Dunn ´s Method, *P* > 0.05, *P* < 0.05, <0.01. **e** Representative spatial heatmaps for naïve and Cort-mice during a social recognition task. Color code denotes accumulated time. **f** Behavioral analysis of social preference for naïve and Cort-mice both implanted with the fiber photometry cannula (FP; naïve = 14, Cort = 15), and mice without surgical manipulations (Control, naïve = 14, Cort = 8), showing similar performance. Note that cannula implantation did not alter social preference. Cort-mice did not display preference for social interaction. Two Way ANOVA, Holm-Sidak method. *P* > 0.05. Y-axis represents the time spent exploring either the unfamiliar mouse (orange) or object (gray). **g** Representative astrocytic Ca^2+^ activity recordings from fiber photometry in naïve and Cort-mice during a social recognition task. Shaded areas indicate interaction bouts with unfamiliar mouse (orange) or object (gray), and dots indicate astrocyte Ca^2+^ events. **h** Z-score of astrocytic Ca^2+^ response during mouse (continuous line) and object (dashed line) interaction bouts for naïve (*n* = 7) and Cort-mice (*n* = 6). Events were aligned to the exploration onset. Reduced amplitude of Ca^2+^ signals upon mouse interaction was found in Cort-mice (n_Cort_=26, n_naïve_ = 34 Ca^2+^ events). One Way ANOVA, Dunn ´s Method, *P* = 0.031. The center line in BW plots indicates the median, the top and bottom edges indicate the 25th and 75th percentiles, respectively, and the whiskers extend to the maximum and minimum data points. *P < 0.05, **P < 0.01, ***P < 0.001.

For behavioral testing, spontaneous Ca^2+^ signals were analyzed in the open field test (OF), and mice showing <2 events in OF were removed from the analysis. Spontaneous Ca^2+^ events detected during the first 5 min were selected and analyzed. For social recognition test, analysis was restricted to the first 5 explorations for both the neutral object and unfamiliar mouse, to avoid the exponential decay shown after several explorations [65]. To analyze Ca^2+^ events during explorations, events whose peak occurred in the interval defined from 1 s before exploration onset up to 3 seconds after the end of an exploration were selected. To compare across subjects, signals were Z-score transformed, by computing the ratio of the ΔF/F_0_ signal over the standard deviation of the signal during the first 5 min when mice were in the neutral chamber [66]. Ca^2+^ signals were time-aligned from 5 s prior to exploration onset up to 20 s after exploration onset (Fig. 1h). Animal speed was evaluated to discard any possible confounding between astrocytic activity and mouse running speed. For the open field, the mean velocity during the entire duration of each Ca^2+^ event was computed. Then, a linear regression model linking Ca^2+^ event amplitude and mean velocity was fitted using the fitlm function in MATLAB. For the social recognition test, the mean speed associated to each Ca^2+^ event was computed as described above, and only events associated to the first 5 explorations (object and mouse) were considered for downstream analysis. Then, a linear regression was fitted separately for Ca^2+^ events associated to object explorations and for events associated to mouse exploration.

### In vivo serotonergic recordings and analysis

Mice were injected with either AAV5-CAG-flex-iSeroSnFR + AAV5/GFAP-mcherry-cre, or AAV5-GFAP-eGFP-WPRE-hGH virus in the right hemisphere of mPFC, followed by implantation of fiber-optic cannula (fiber core Ø of 400 µm; 0.50 NA; ref. FP400URT Thorlabs) following the same surgical procedure as for GCaMP Fiber Photometry. In addition, AAV9-hSyn-ChrimsonR-tdTom was injected in DRN, and fiber-optic cannula implanted (fiber core Ø of 400 µm; 0.50 NA; ref. FP400URT, Thorlabs). In total, 2–3 weeks after surgery, behavioral testing started to allow for viral expression and animal recovery. Fiber photometry recordings were performed using FPS_1S_GCaMP system. A 465 nm LED delivered at 70–130 µW was used for iSeroSnFR excitation, and a 590 nm LED (M590F3 - 590 nm, Fiber-Coupled LED, 1000 mA, SMA- LEDD1B - T-Cube LED Driver) at 5 mW was used for optogenetic stimulation of DRN. iSeroSnFR and eGFP signals were recorded while the mouse was freely moving in the OF arena. Each animal underwent between 2 and 6 trials of DRN stimuli (40 Hz, 10 s) with 50 s inter-intervals, with 3 min of baseline recordings previous to DRN stimulation. For the analysis, the first min of recordings was discarded to account for signal photobleaching effects. Photometry signals were collected interleaved at a sampling frequency of 50 Hz and analyzed as described above. Signals were low-pass filtered with a Chebyshev Type II filter with a 30 Hz cut-off frequency. ΔF/F_0_ signal was computed for iSeroSnFR and eGFP fluorescence measurements. For traces representation (Extended Data Fig. 5e, g), signals were low-pass filtered with a cut-off frequency of 20 Hz to reduce noise, and Z-score was computed to compare across subjects. Analysis was restricted to the signals recorded 30 s before (baseline) and the 30 s after the stimulation onset.

### Ex vivo electrophysiological recordings

Whole-cell patch-clamp recordings from layer 2/3 pyramidal neurons and astrocytes of mPFC were performed. Neuronal currents were recorded by borosilicate capillaries (3-6 MΩ) filled with an intracellular solution that contained [in mM]: K-gluconate 135, KCl 10, HEPES 10, MgCl_2_ 1, and ATP-Na_2_ 2 (pH = 7.3). In some experiments, intracellular solution was modified containing GDPβS 2 mM. Astrocytic whole-cell recordings were performed (8-10 MΩ) using an intracellular solution containing [in mM]: BAPTA-K_4_ 40, NaCl 8, MgCl_2_ 1, HEPES 10, GTP-tris salt 0.4 and ATP-Na_2_ 2 (pH = 7.3). Astrocyte recordings lasted ≥ 30 min to allow the dialysis of BAPTA through the gap-junction connected astrocytic network [67]. Recordings were obtained with PC-ONE amplifiers (Dagan Corporation, Minneapolis, MN) in voltage-clamp conditions and the membrane potential was held at –70 mV. Series and input resistances were monitored throughout the experiment using –5 mV pulses. Recordings with access resistance change >20% were rejected. Signals were fed to a Pentium-based PC through a DigiData 1440 interface board (Axon Instruments). Signals were filtered at 1 kHz and acquired at 10 kHz sampling rate. The pCLAMP 10.7 software (Axon Instruments) was used for stimulus generation, data display, acquisition, and storage. Experiments were performed at RT.

Slow inward currents (SICs) were recorded in the presence of TTX (1 µM) and distinguished from miniature synaptic currents (mEPSCs) by their slower time courses [68, 69]. SICs were abolished by the presence of AP5, a selective antagonist of NMDARs (50 μM; 0.27 ± 0.04, *n* = 35 in control vs 0.05 ± 0.02, *n* = 13 in AP5; One Way ANOVA, Dunn´s Method, *P* < 0.001. Source data) [68]. Excitatory postsynaptic currents (EPSC) were elicited by theta capillaries (2-5 μm tip diameter) located in layer V and filled with aCSF. Paired pulses (250 μs duration; 75 ms interval) were continuously delivered at 0.33 Hz by stimulator S-900 (Dagan Corporation). Baseline of synaptic activity was measured 5 min before local application of 5-HT/CNO. aCSF included picrotoxin (50 μM) to block GABA_A_-dependent inhibitory synaptic activity. For the analysis, neuronal recordings that did not show stable responses were discarded. Recordings that did not last >25 min were excluded from the delayed responses quantification, but considered for short responses analysis.

To monitor depolarization or hyperpolarization of neuronal membranes induced by puff application, changes in holding current (HC) were recorded and computed as HC index: [HC (i) - HC (baseline)]/absolute value [HC (i) + HC (baseline)]. i =HC value at different time points after puff application, baseline= mean HC before puff application.

### Optogenetic stimulation

Light stimulation with the CoolLED illumination system, 550 nm light pulses of 50 ms at 5 Hz (1 mW) was applied for the electrophysiological recordings in mPFC slices, which activated ChrimsonR-expressing fibers and induced the endogenous release of 5-HT. For ex vivo astrocyte Ca^2+^ recordings, 640–660 nm light stimulation (10 s, continuous light, <1 mW) through external laser was used to activate ChrimsonR-expressing fibers.

### Immunohistochemistry and confocal microscopy

Mice were euthanized by sodium pentobarbital i.p. injections and transcardially perfused with phosphate-buffered saline (PBS: 137 mM NaCl, 2.7 mM KCl, 10 mM Na_2_HPO_4_, 2 mM KH_2_PO_4_, pH 7.4; 15714 Electron Microscopy Sciences, EM Grade) followed by ice-cold 4% paraformaldehyde (PFA). Brains were removed and postfixed overnight (o/n) at 4 °C in 4% PFA. Coronal brain slices (50 μm thick) were obtained with a VT1000S vibratome (Leica) and collected as floating sections. For immunostaining, slices were first washed with PBS and permeabilized with 0.2% Triton/PBS. Nonspecific binding was blocked with PBS containing 1–5% goat serum and 0.3% Triton-X 100 for 1 h. Samples were then incubated with the corresponding primary antibodies in blocking solution overnight at 4 °C: rabbit anti-S100-β (1:200, Abcam, Cambridge, UK; RRID: AB_306716), mouse anti-SERT (1:500, Synaptic Systems CI.64G6), mouse anti-Neuronal Nuclei (NeuN, 1:500, Merck, MAB377), rabbit anti-5HT2AR (1:100, Immunostar, 24288). After three 20-min washes in blocking solution at RT, floating sections were incubated for 1 h RT with specific secondary antibodies: Alexa Fluor 488 (goat anti-mouse; 1:200, Bioss Inc., Woburn, MA, RRID:AB_10892893); Alexa Fluor 647 (goat anti-rabbit; 1:200, Thermo Fisher Scientific, RRID:AB_2535813); Alexa Fluor 488 (goat anti-rabbit; 1:200, Thermo Fisher Scientific, RRID:AB_143165); Pacific blue (goat anti-mouse 1:200, Thermo Fisher Scientific, RRID:AB_10374586). After three 20-min of PBS washes containing 0.1% Triton X-100, slices were incubated with DAPI (1.5 μg/mL, Sigma-Aldrich) for 10 min. Finally, sections were washed, three times 20-min each, in PBS and mounted with Vectashield antifade mounting medium (H-1000, Vector Laboratories, Burlingame, CA), and images acquired using a Leica SP-5 confocal microscope (Leica Biosystems). Quantification was performed using Fiji software (ImageJ 1.53i, NIH). All the antibodies used in the study have been satisfactorily validated by commercial vendors.

Co-localization of constructs encoded by viral vectors (cyto-GCaMP6f and ChrimsonR-tdTom) with astrocytic, neuronal markers and serotonergic labeling was performed. Maximal projections of z-stacks (10 μm thickness) obtained with a 40× 1.25 NA oil immersion objective (single optical sections 2 μm) for GCaMP6f images, and 63× 1.40 NA oil immersion objective (single optical sections 1 μm) for ChrimsonR-tdTom images were used (Leica SP-5). After thresholding the GCaMP6f or ChrimsonR-tdTom image a mask was created. This mask was superimposed over the astrocytic marker S100β, neuronal marker NeuN or serotonergic projections labeled with anti-SERT image, and ROIs were automatically (ChrimsonR-tdTom image) and manually (GCaMP6f image) detected and measured. 5 background ROIs were manually selected in the S100β, NeuN and anti-SERT image. Colocalization was considered when the mean intensity value of S100β, NeuN and anti-SERT ROIs was above the background average plus 3 times the standard deviation of the signal. Positive ROIs were tagged with 1, whereas negative ROIs were tagged with 0, and the percentage of positive ROIs was calculated for each field of view.

The presence of 5-HT2AR puncta in mPFC astrocytes was determined using Fiji. Maximal projection of 3 μm of thickness was obtained from images acquired with 40× 1.3 NA oil immersion objective in Stellaris 8 STED (Leica). Astrocyte somata and processes were manually identified by endogenous EGFP labeling in *Aldh1/1*-EGFP mice using the ROI manager tool. The number of puncta within identified *Aldh1/1*-EGFP astrocytes was assessed using the process Find Maxima in Fiji software, stablishing the prominence value as the mean background fluorescence plus 5 times the standard deviation. Astrocytes were considered positive for 5-HT2AR expression when at least 4 puncta were detected.

### Behavioral assays

Handling period was performed for 5 min during 5 consecutive days before behavioral testing, which started 3 days after ending Cort-treatment. Mice were transferred to the testing room for at least 30 min before the experiment to reduce stress [70]. All tasks were performed between 08:00 am and 3:00 pm. Arenas and maze were cleaned with a 0.1 % acetic acid dissolved in water between the sessions.

#### Forced swimming test (FST)

FST was performed in a clear acrylic cylinder (29 cm height, 12 cm diameter) filled with warm water (22–23 °C). Mouse behavior was video-recorded for 6 min. Immobility score was analyzed during the last 4 min using EthoVision XT 7 software (Noldus Information Technology, Inc.; Leesburg, VA). Time when mice were immobile was used as indicator of hopelessness, which has been related with depressive-like phenotypes in rodents [71, 72].

#### Elevated plus maze (EPM) test

EPM is used as a reference of mouse anxiety levels [73]. The maze had two closed and two open arms (30 × 10 × 5 cm each) and is placed 1 m above the ground. At the beginning of the session (5 min total duration), the animal was placed at the intersection of the arms. The time spent in the open and enclosed arms was recorded by EthoVision XT 7 software. The exploration index was computed as the time spent in open arms vs the total time spent in open and closed arms. An entry was considered when the mouse had all four paws inside the arm of the maze.

#### Object in place (OIP) test

Acrylic open field arena (40 × 40 × 40 cm) with 4 non-identical objects placed near the corners of the arena [74] was used. Mouse behavior was recorded by EthoVision XT 7 software. In total, 3 days prior testing, animals were individually habituated to explore the empty arena for 30 min. In the first trial, animals were allowed to freely explore the different objects for 5 min. After 5 min of inter-trial delay, mice were allowed to explore for 3 min the arena where two objects were reallocated (new object location). The index was computed as the time spent exploring the objects in novel locations of the total time exploring. Exploration was defined as reaching the object with the nose.

#### Novel object recognition (NOR) test

NOR test was conducted using the same arena as OIP. Once mice were habituated to the arena, they were allowed to explore two identical objects for 5 min. After one hour, mice are reintroduced into the arena where an object has been replaced by a novel one [75]. The index was computed as the total time spent exploring novel object versus the total time of exploration (novel + familiar).

#### Open field (OF) test

OF test was conducted using the same arena as OIP and NOR. Once mice were habituated to the arena, they were allowed to move freely in the arena for 5 min. Movement cumulative duration time (s) was recorded by EthoVision XT 7 software.

#### Social recognition (SR) test

Sociability measurements were performed in a clear acrylic three-chamber cage (60 × 42 × 20 cm each) [76, 77]. The middle chamber was used as a resting point, and the chambers on the side hold two small cylindrical cages that contained an unfamiliar mouse or a neutral small object [78, 79]. Unfamiliar male mice were habituated to remain into the cylinder cages 2 days prior testing. On the testing day, mice were placed in the middle chamber (to prevent access to the side chambers, clear acrylic sliding doors were used), and were allowed to explore it for 5 min. Afterwards, the doors were opened and the animal was able to freely explore the “social chamber” (holding the unknown mouse) or the “non-social chamber” (holding the object) for 10 min. An exploration was considered when mouse’s nose was in contact with the cage.

For CNO or AIDA experiments, i.p. injections were conducted 20–30 min before the beginning of each task. Saline (vehicle) was i.p. injected as control in a subset of mice. Animals with no exploratory behavior in a particular test were eliminated from the analysis of that test.

### Drugs and chemicals

The following reagents were bath-applied during ex vivo recordings for at least 15 min before testing: picrotoxin (50 µM, Sigma, Cat#P1675; CAS:124-87-8), D-AP5 (50 µM, Tocris, Cat#0106; CAS: 79055-68-8), LY367385 (100 µM, Tocris, Cat#1237; CAS: 198419-91-9), SB 216641 hydrochloride (50 µM, Tocris, Cat#1242; CAS 193611-67-5) AM251 (2 µM, Tocris, Cat#1117; CAS: 183232-66-8), MRS 2179 tetrasodium salt (10 µM, Tocris, Cat#0900; CAS: 1454889-37-2), CGP 55845 hydrochloride (5 µM, Tocris, Cat#1248; CAS 149184-22-5), Ketanserin tartrate (10 µM, Tocris, Cat#0908 CAS 83846-83-7), RS127445 hydrochloride (1 µM, Tocris, Cat#2993 CAS 199864-86-3), RS102221 hydrochloride (1 µM, Tocris, Cat#1050 CAS 187397-18-8), MDL100907 (1 μM; Tocris, Cat#4173 CAS 139290-65-6), WAY100135 (10 μM; Tocris,Cat#1253 CAS149007-54-5), tetrodotoxin (TTX, 1 µM, Alomone labs, Cat#T-550; CAS: 18660-81-6). A constant flow of fresh aCSF plus selected drugs was continuously perfused into the recording chamber. Serotonin hydrochloride (1 mM, Tocris, Cat# 3547 CAS 153-98-0), Clozapine N-oxide (1 mM, Tocris, Cat# 4936 CAS 34233-69-7), and Adenose 5’triphosphate disodium salt hydrate (ATP, 1 mM, Sigma-Aldrich, Cat# A7699) were locally applied by a micropipette. The following inhibitors were added to the intracellular solutions: 1,2-bis(2-aminophenoxy)ethane-N,N,N′,N′-tetraacetic acid (BAPTA, 40 mM, Sigma-Aldrich, Cat# A4926), and guanosine 5′-[β-thio]diphosphate (GDPβS), trilithium salt (2 mM, Merck, Cat# G7637) 1-aminoindan-1,5-dicarboxylic acid (AIDA, 5 mg/kg, Tocris, Cat# 0904 CAS 168560-79-0) and Clozapine N-oxide (3 mg/kg, Tocris, Cat# 4936 CAS 34233-69-7) were administered via i.p. Fluo-4 AM (Invitrogen, Cat# F14201), Pluronic® F-127 (Merck, Cat# P2443), Dimethyl sulfoxide (DMSO, Sigma-Aldrich, Cat# D8418).

### Statistical analysis

All animal samples and biological replicate numbers in this study are in line with well-accepted standards from the literature for each method. All data presented in this work were obtained from experimental replicates; that is, multiple animal cohorts from different litters, at least three experimental repeats for each assay, and production of biological replicates. All attempts of replication were successful. Each statistical test was used according to the design of the experiment and the structure of the data. According to the normality of the data distribution, two-group comparisons were performed using One Way ANOVA, Kruskal-Wallis on ranks, One-way ANOVA with Dunn´s method, Tukey test and Holm-Sidak post-hoc analysis; or paired *T* test or Wilcoxon matched-pair tests, respectively. In experiments involving several conditions, Two-way ANOVA was performed followed by post-hoc test (Holm-Sidak method). No statistical methods were used to predetermine sample sizes in this study, which were determined according to the accepted practice for the applied assays [9, 80]. Experiments, except the behavioral test, were not performed with blinding to the conditions of the experiments. However, data analyses were performed blinded to the scorer or did not require manual scoring. Descriptive statistics are reported as the mean ± s.e.m., and box and whisker plots. In BW plots the central mark indicates the median, and the bottom and top edges of the box indicate the 25th and 75th percentiles, respectively. The whiskers extend to the maximum and minimum data points (not considered outliers). In scatter dot plot graphs, the central mark indicates the median, and the top and bottom edges, so-called range, correspond to the maximum and minimum values, respectively. Statistically significant differences were established at *P < 0.05, **P < 0.01 and ***P < 0.001, two-sided.

## Results

### Abnormal astrocyte Ca^2+^ dynamics in depressive-like behaviors

Chronic stress and activation of the HPA axis can induce a variety of behavioral responses that parallel depressive symptoms [15]. Additionally, mice treated via oral exposure to the stress hormone corticosterone (Cort) recapitulate the anhedonic- and helplessness-like behaviors. This in turn, is reversible by chronic antidepressant treatment, being considered as a model of stress-induced depressive-like behaviors in rodents [29–31, 33]. Here, we used chronic Cort treatment in juvenile mice (Cort-mice) to evaluate the impact of chronic stress on astrocyte Ca^2+^ signaling. Animals stressed in early childhood show increased anxiety-like behavior [81], decreased spatial memory [82] and social impairments [83]. Then, we first confirmed that oral consumption of Cort induced increased levels of hair corticosterone deposits (cf. [56]) without affecting the corticosterone serum levels [33, 55], reduced body weight gain [84], and longer latencies of immobility in the forced swimming test (FST), which supported the efficacy of Cort-treatment to induce depressive-like behaviors in young mice (Fig. 1a, Extended Data Fig. 1a, b) [33, 55]. Next, we analyzed the impact of this treatment on astrocytic Ca^2+^ signals, recording Ca^2+^ activity in vivo at mPFC in naïve and Cort-mice (Fig. 1). Viral injections of Ca^2+^ sensor (AAV5-GFAP-cytoGCaMP6f) were performed in both phenotypes and fiber optic were implanted at mPFC to monitor astrocytic Ca^2+^ dynamics (Fig. 1b, c). In total, 2–3 weeks later, both spontaneous Ca^2+^ events and behavioral-driven responses were evaluated. In the open field test (OF), Cort-mice displayed faster astrocyte Ca^2+^ signals (17.39 ± 0.81 s Cort vs 20.57 ± 0.95 s naïve, n_Cort_ = 90 events, *n* = 6 mice; *n*_naïve_ = 60 events, n = 7 mice; One Way ANOVA, Dunn´s Method, *P* = 0.007), resulting in a reduced inter-event interval (0.53 ± 0.08 min Cort vs 0.88 ± 0.12 min naïve; One Way ANOVA, Dunn´s Method, *P* = 0.017), with an increased amplitude compared to the control group (0.03 ± 0.004 ΔF/F_0_ Cort vs 0.02 ± 0.003 ΔF/F_0_ naïve; One Way ANOVA, Dunn´s Method, *P* = 0.047) (Fig. 1c, d). Astrocyte resting Ca^2+^ levels did not show significant differences between naïve (F_0_: 0.90 ± 0.17, *n* = 7) and Cort-mice (F_0_: 1.10 ± 0.13, *n* = 6; One-Way ANOVA, *P* = 0.388). These data suggest that astrocytes display dysfunctional intracellular Ca^2+^ signaling under depressive-like conditions. Since depression has an important impact on social behaviors [85], we confirmed that Cort-mice showed such social impairments in the three-chamber social test (Fig. 1e, f) [86]. We recorded astrocytic Ca^2+^ during the social test and analyzed the Ca^2+^ events when mice explored the inanimate object or the caged mouse (Fig. 1g). Interestingly, Cort-mice showed reduced astrocytic Ca^2+^ activity during mouse explorations compared to the control group (Fig. 1h). While astrocytic Ca^2+^ events during the explorations of the inanimate object were similar in both groups (1.42 ± 0.36 Cort vs 1.38 ± 0.26 naïve, n_Cort_ = 29 events, *n* = 6 mice; *n*_naïve_ = 35 events, *n* = 7 mice; One Way ANOVA, Dunn´s Method, P = 0.471), Ca^2+^ fluorescence signals during social interactions were decreased in Cort-mice (1.12 ± 0.23 Cort vs 3.26 ± 0.69 naïve, n_Cort_ = 26, n_naïve_ = 34 events, One Way ANOVA, Dunn´s Method, *P* = 0.031) (Fig. 1h). Additionally, the relationship between animal running speed and Ca^2+^ events amplitude was analyzed in order to evaluate any possible confounding between astrocytic activity and locomotion [87–89]. No significant correlation was found between those parameters, suggesting a non-direct effect of locomotion to the population astrocytic Ca^2+^ signals in mPFC (Extended Data Fig.1c, d). These results indicated that depressive-like behavior induced a robust impact on the intracellular Ca^2+^ in mPFC astrocytes both for baseline activity and social-engaged behaviors.

### Altered serotonergic-driven astrocytic Ca^2+^ responses in depressive-like states

**Fig. 2:**
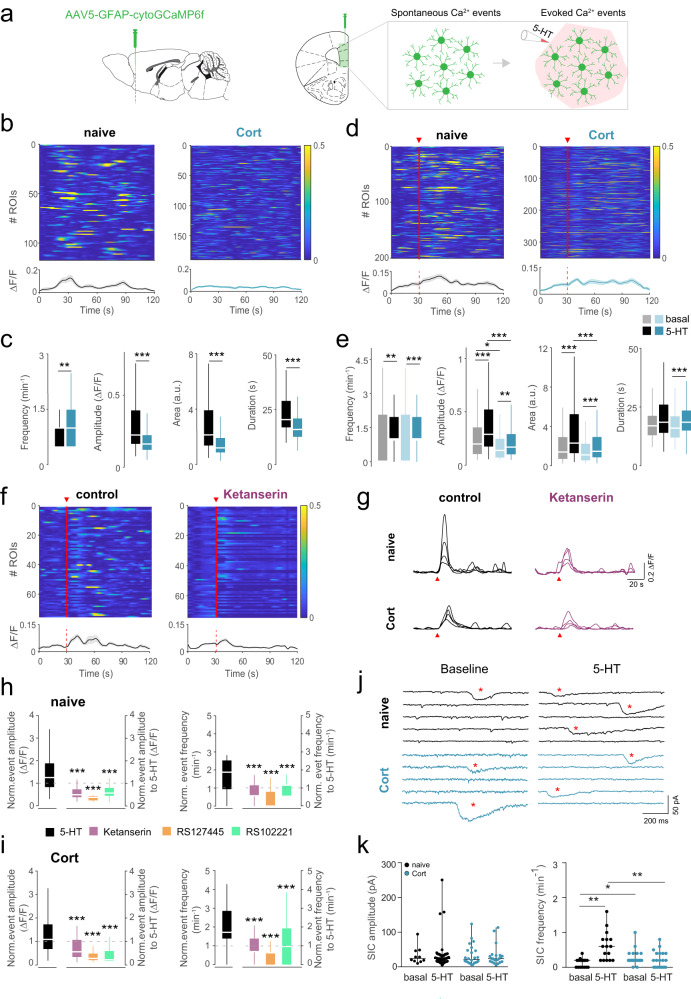
Spontaneous and 5-HT-evoked astrocyte Ca^2+^ signaling in naïve and Cort-mice. **a** Left. Sagittal and coronal scheme of viral injection in the mPFC (green shaded area) to target astrocytes using AAV5-GFAP-GCaMP6f virus. *Right*. Squeme of spontaneous and 5-HT-evoked calcium events in mPFC astrocytes. **b** Heatmaps of spontaneous ROIs activity and average population activity in astrocytes of naïve (black, *n* = 117 ROIs, *n* = 3 mice) and Cort-mice (blue, *n* = 190 ROIs; *n* = 2 mice). Data are presented as mean ± s.e.m. Color code denotes fluorescence changes. **c** Box and whisker (BW) plots representing the dynamics of Ca^2+^ astrocytic events in naïve and Cort mice. Cort-mice showed an increased frequency of Ca^2+^ events (*P* = 0.006) but decreased amplitude, area and duration of events. One Way ANOVA, Dunn’s Method, *P* < 0.001. **d** Heatmaps of 5-HT evoked ROIs activity and average population activity in astrocytes of naïve (*n* = 200, *n* = 3 mice) and Cort-mice (*n* = 350, *n* = 2 mice). Red triangle and bar denote 5-HT puff application (1 mM, 10 s, 1 bar). **e** BW plots representing the dynamics of Ca^2+^ astrocytic events in response to 5-HT of naïve and Cort-mice. Reduced amplitude and area of Ca^2+^ events were found in Cort-mice (black bars vs dark blue bars). One Way ANOVA, Dunn’s Method, *P* < 0.001. **f** Heatmaps of 5-HT evoked ROIs activity and average population activity in astrocytes of naïve mice, in basal conditions (left)and in presence of ketanserin (right, *n* = 75, *n* = 2 mice). **g** Representative traces of Ca^2+^ astrocytic signals from naïve and Cort-mice evoked by local 5-HT puff (red triangle) in control conditions (left, black) and after bath application of ketanserin (right, purple). **h**, **i** BW plots representing changes in amplitude and frequency of Ca^2+^ events induced local 5-HT stimulation before (black) and after bath application of 5-HT2R family antagonists (ketanserin, purple; RS127445, orange, *n* = 70, *n* = 2 mice; RS102221, green, *n* = 69, *n* = 2 mice) in naïve mice (**h**), and Cort-mice (ketanserin, *n* = 47, *n* = 2 mice; RS127445, *n* = 70, *n* = 2 mice; RS102221, *n* = 51, *n* = 2 mice) (**i**). Ca^2+^ event amplitude and frequency induced by 5-HT was normalized to spontaneous activity before 5-HT stimulation (black bars). These parameters were normalized to the Ca^2+^ signals evoked by 5-HT before 5-HT2R antagonists family blockade. Note the reduced activity under the influence of each antagonist. *P* < 0.001. One Way ANOVA, Dunn’s method. **j** Representative traces of slow inward currents (SICs) before and after local 5-HT application (1 mM, 10 s, 1 bar) in naïve and Cort-mice. Red asterisk indicates the presence of SICs. **k** Scatter plot of SICs recorded before (5 min) and after 5-HT (5 min) showing a significant increase in frequency after 5-HT stimulation in naïve mice (*n* = 16 cells, *n* = 2 mice; Tukey Test, *P* < 0.001), without further changes in current amplitude for both naïve and Cort-mice (*n* = 19 cells, *n* = 3 mice). One Way ANOVA, Dunn’s Method, *P* = 0.758 for naïve, *P* = 0.982 for Cort. Note the enhanced SICs frequency in resting conditions for Cort-mice, which were insensitive to further increase by 5-HT stimulation. One Way ANOVA, Dunn’s Method, *P* = 0.033. Data shown as median ± range (min and max values). The center line in BW plots indicates the median, the top and bottom edges indicate the 25th and 75th percentiles, respectively, and the whiskers extend to the maximum and minimum data points. *P < 0.05, **P < 0.01, ***P < 0.001.

Dysfunctions of the 5-HT system are involved in mood disorders, including depression [90]. Since astrocytes express transporters and receptors for the sensing of serotonergic transmission [91], we continued by analyzing the astrocytic Ca^2+^ responses induced by 5-HT local stimulation in naïve and Cort-mice in mPFC brain slices. Following a similar viral strategy, AAV5-GFAP-cytoGCaMP6f was selectively expressed in mPFC astrocytes and local Ca^2+^ events were monitored in basal conditions and in response to local 5-HT application (1 mM, air puff 10 s, 1 bar) (Fig. 2a). Basal activity recordings in presence of TTX (1 μM) displayed significant differences between astrocytes from naïve and Cort-mice. Faster Ca^2+^ events (17.33 ± 0.45 s Cort vs 22.95 ± 0.77 s naïve, n_Cort_ = 190 ROIs; n_naïve_ = 117 ROIs, *n* = 2 Cort-mice, *n* = 3 naïve mice; One Way ANOVA, Dunn´s Method, *P* < 0.001) with reduced amplitude (0.19 ± 0.01 ΔF/F_0_ Cort vs 0.36 ± 0.03 ΔF/F_0_ naïve; One Way ANOVA, Dunn´s Method, *P* < 0.001) were found in Cort-mice (Fig. 2b, c). The analysis of astrocytic resting Ca^2+^ levels did show an enhancement of F_0_ values in Cort-mice (94,48 ± 2,07, *n* = 190) compared with naïve mice (74,68 ± 1,95, *n* = 117; One-Way ANOVA, *P* < 0.001). In addition, mPFC astrocytes from Cort-mice showed higher frequencies of Ca^2+^ events (1.03 ± 0.04 s Cort vs 0.85 ± 0.04 s naïve; One Way ANOVA, Dunn´s Method, *P* = 0.006) (Fig. 2c), in line with the abnormal Ca^2+^ dynamics found in in vivo recordings. Next, we found that local application of 5-HT was able to engage Ca^2+^ signaling in mPFC astrocytes in both conditions (Fig. 2d, e), but Cort-mice showed a reduced amplitude in 5-HT-driven astrocyte Ca^2+^ events (0.28 ± 0.02 ΔF/F_0_ Cort vs 0.40 ± 0.03 ΔF/F_0_ naïve; One Way ANOVA, Dunn´s Method, *P* < 0.001) (Fig. 2e). Remarkably, in contrast to 5-HT-mediated signaling, Cort-mice showed enhanced astrocytic Ca^2+^ events induced by ATP (1 mM, 10 s, 1 bar), a potent inductor of astrocytic Ca^2+^ elevations [92], compared to naïve astrocytes (1.17 ± 0.08 ΔF/F_0_ Cort-mice vs 0.89 ± 0.05 ΔF/F_0_ naïve mice; One Way ANOVA, Dunn’s Method, *P* = 0.006; Extended Data Fig. 2a, b). These results confirmed the dysfunctional astrocyte Ca^2+^ signaling in Cort-mice and the selective downregulation of 5-HT-driven astrocytic responses.

Ca^2+^ signaling has been associated with the release of active substances from astrocytes, such as glutamate, d-Serine, ATP among others [1, 2, 93], which impact both functional and structurally synaptic activity and behavior [94, 95]. Astrocytes can stimulate neuronal NMDA receptors activation inducing slow inward currents (SICs) and modulating neuronal excitability [68, 96]. Here, we analyzed the ability of mPFC astrocytes to induce SICs in principal cells from layer 2/3 in mPFC in naïve and Cort conditions. SICs were recorded in basal conditions in both naïve and Cort-mice (Fig. 2j), but only naïve mice displayed a significant increase after 5-HT stimulation (0.61 ± 0.10 min^-1^ naïve vs 0.24 ± 0.05 min^-1^ Cort., n_naïve_ = 16 SICs, *n* = 2 mice; n_Cort_ = 19 SICs, *n* = 3 mice; One Way ANOVA, Dunn´s Method, *P* = 0.002) (Fig. 2k). Additionally, Cort-neurons showed an elevated SIC frequency in resting conditions (0.27 ± 0.05 min^–1^ Cort. vs 0.13 ± 0.03 min^–1^ naïve; One Way ANOVA, Dunn´s Method, *P* = 0.033), which might correlate with the increased frequency of Ca^2+^ events found in astrocytes from Cort-mice (Fig. 2c). These data suggest that not only the spontaneous activity, but also 5-HT-engaged astrocyte Ca^2+^ signaling and gliotransmission were compromised in depressive-like behaviors.

Among the different receptors of the 5-HT system, astrocytes from mPFC express 5-HT2 receptors, including 2 A, 2B and 2 C, which are coupled to IP3 intracellular signaling [97–99], one of the main routes to trigger intracellular Ca^2+^ elevations in astrocytes [100]. Thus, we evaluated the role of these receptors in the 5-HT-driven astrocyte responses (Fig. 2f–i). Blockade with selective antagonists for the different 5-HT2 receptor subtypes, ketanserin for 5-HT2A, RS127445 for 5-HT2B and RS102221 for 5-HT2C, resulted in a significant reduction of both the amplitude and frequency of astrocyte 5-HT-driven Ca^2+^ events in control and Cort-mice (Fig. 2f–i), indicating that Cort-treatment involved important changes in astrocyte Ca^2+^ dynamics in mPFC affecting spontaneous, but also 5-HT-engaged signaling. The expression of 5-HT2A in astrocytes was evaluated by immunohistochemical studies in *Aldh1/1*-EGFP mice [101] (Extended Data Fig. 3a). These results support at least 5-HT2A receptor expression in cortical astrocytes and further confirm the functional evidences shown by Ca^2+^ imaging. Ketanserin has been described to block 5-HT2C receptors [102], which might cover the selective actions of 5-HT2ARs to the astrocytic Ca^2+^ signaling. Then, a more selective 5-HT2A antagonist MDL100907 (1 μM) was used (Extended Fig. 3b–d), which corroborated the role of 5-HT2ARs in the 5-HT-driven astrocyte Ca^2+^ signals. In addition, the dependence of IP3-engaged intracellular signaling in astrocytes by 5-HT was evaluated and Ca^2+^ recordings were performed in *Ip3r2*^*−/−*^ mice, which show downregulated Ca^2+^ signaling in astrocytes [100]. As expected, 5-HT stimuli did not evoke significant Ca^2+^ changes (Extended Data Fig. 3e, f). Finally, to ensure the main role of 5-HT2Rs to the 5-HT-driven astrocyte Ca^2+^ responses the contribution of other 5-HTRs, e.g., 5-HT1A, was evaluated. The presence of WAY100135 (10 μM), a selective antagonist of 5-HT1A, did not affect the 5-HT induced Ca^2+^ signals in cortical astrocytes (Extended Data Fig. 3g, h), supporting the relationship between activation of 5-HT2R family and astrocyte Ca^2+^ signaling.

Depression behavior has been associated with low concentrations of 5-HT in different brain areas [103–105]. Hence, we next investigated whether the reported abnormal Ca^2+^ responses in astrocytes induced by Cort-treatment might be related to reduced levels of 5-HT in mPFC. To do so, we evaluated the ability of mPFC astrocytes to sense the endogenous release of 5-HT from serotonergic brain areas, such as DRN. First, via ex vivo recordings in naïve mice, we found that 45.9% of the recorded mPFC astrocytes showed robust Ca^2+^ responses after selective light stimulation of DRN afferents (550 nm light pulses of 50 ms at 5 Hz 1 mW) expressing the excitatory opsin ChrimsonR (AAV9-hSyn-ChrimsonR-tdTom) targeting mPFC (Extended Data Fig. 4a–d); confirming that DRN neuronal activity can stimulate Ca^2+^ signaling in mPFC astrocytes (Extended Data Fig. 4d, e). Next, by in vivo recordings, the endogenous release of 5-HT was estimated by using 5-HT (GRAB_5-HT_/iSeroSnFR) sensor [106] expressed specifically in mPFC astrocytes both in naïve and Cort-mice. Viral injections were performed using a combination of AAV5-GFAP-mCherry-cre + AAV5-CAG-flex-iSeroSnFR for selective GRAB_5-HT_ expression in mPFC astrocytes, while AAV9-hSyn-ChrimsonR-tdTom was expressed in DRN neurons (Extended Data Fig. 5). Light stimulation of DRN neurons (40 Hz, 10 s) boosted GRAB_5-HT_ fluorescence changes in mPFC in naïve mice (from 0.22 ± 0.18 to 1.56 ± 0.35, *n* = 3 mice; One Way ANOVA, Holm-Sidak method, *P* = 0.010), while attenuated fluorescence signals were obtained from Cort-mice after DRN stimulation (0.33 ± 0.25 in Cort-mice vs 1.56 ± 0.35 in naïve mice, n_Cort_ = 3, n_naïve_ = 3 mice; One Way ANOVA, Holm-Sidak method, *P* = 0.006) (Extended Data Fig. 5e, f). Altogether, these data not only support the hypothesis that depressive-like behaviors are related with reduced 5-HT levels (cf [103, 104]), but also highlight the capability of astrocytes to sense serotonergic activity far from the serotonergic nuclei.

### Abnormal 5-HT driven synaptic transmission in corticosterone-treated mice

**Fig. 3:**
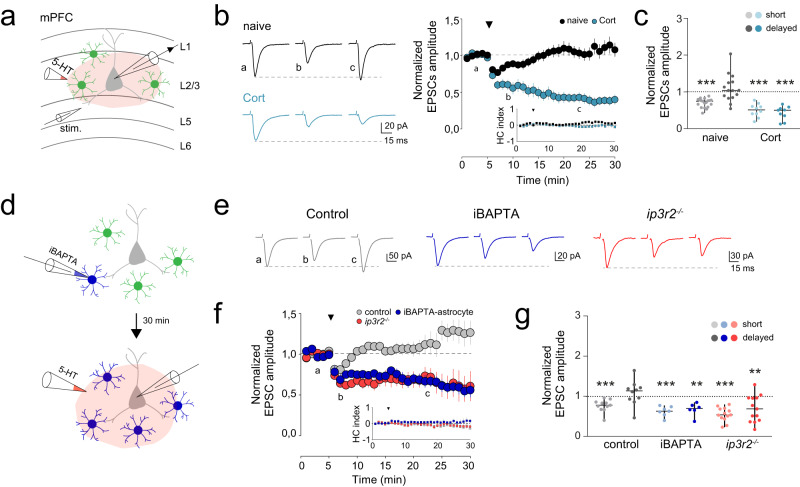
mPFC abnormal 5-HT driven synaptic plasticity in depressive-like conditions. **a** Schematic representation of whole-cell recording of pyramidal neurons in layer 2/3 mPFC slices and neighboring astrocytes, including the glass pipettes for electrical stimulation in layer 5 and local puff of 5-HT in layer 2/3. **b** *Left*, representative EPSC traces (average from 20 consecutive responses) recorded from pyramidal neurons before (**a**), and after (**b**, **c**) 5-HT application in naïve (black) and Cort-mice (blue). Short (**b**) and delayed (**c**) synaptic responses are shown. Right, average of normalized EPSC amplitude and Holding Current (HC) index over time before and after 5-HT stimulus in naïve (*n* = 19 cells, *n* = 8 mice) and Cort-mice (*n* = 10 cells, *n* = 5 mice). Black triangle denotes 5-HT local puff application (1 mM, 10 s, 1 bar). Data shown as mean ± s.e.m. **c** Scatter plot of EPSC amplitude changes analyzed during the first 5 min of 5-HT, for short, and after 25 min for delayed synaptic effects. Note the sustained synaptic depression of EPSCs in Cort mice (*n* = 9 cells); Paired-*t* test, *P* < 0.001. Data shown as median ± range (min and max values). **d** Schematic drawing of intracellular loading of BAPTA into the astrocyte network followed by pyramidal neuron recordings and 5-HT local puff application. **e** Representative EPSC traces (average from 20 consecutive responses) recorded from pyramidal neurons before (**a**), and after (**b**, **c**) 5-HT application in control mice (gray), iBAPTA-astrocyte control mice (blue) and Ip3r2^–/–^ mice (red). Short (**b**) and delayed (**c**) synaptic responses are shown. **f** Average of normalized EPSC amplitude and HC index over time before and after 5-HT stimulus in control mice (*n* = 11 cells, *n* = 7 mice), iBAPTA-astrocyte control mice (*n* = 7 cells, *n* = 5 mice) and Ip3r2^–/–^ ﻿mice (*n* = 13 cells, *n* = 10 mice). **g** Scatter plot of EPSC amplitude changes analyzed during the first 5 min of 5-HT, for short, and after 25 min for delayed synaptic effects. Note that reduced astrocyte calcium activity induced a persistent synaptic depression, *P* = 0.004 for iBAPTA-astrocyte. *P* = 0.006 for *Ip3r2*^*–/–*^ mice. Paired *t* test. **P < 0.01, ***P < 0.001.

It has been reported that depressive-like states disrupt excitatory synaptic transmission and synaptic plasticity [107]. Synaptic transmission and plasticity are tightly regulated by serotonergic signaling [108], which is related to important physiological processes, such as sleep, body temperature, appetite, pain and motor activity [109]. Next, we further analyzed the impact of Cort-treatment on 5-HT-driven synaptic responses in mPFC. Layer 2/3 is the main intracortical processing layer of the prefrontal cortex, being sensitive to stress and psychiatric diseases [110, 111]; therefore, excitatory synaptic transmission (EPSCs) was recorded from layer 2/3 principal cells of mPFC brain slices (Fig. 3a, Extended Data Fig. 6). Local application of 5-HT (1 mM, 10 s, 1 bar) evoked a transient depression of EPSCs in control conditions (0.70 ± 0.03, *n* = 19 cells, *n* = 8 mice; Paired *t* test, *P* < 0.001) (Fig. 3b, c) (cf. [112]), which was confirmed by endogenous release of 5-HT after selective stimulation of DRN projections to mPFC by viral expression of ChrimsonR (Extended Data Fig. 4f, g). Indeed, light activation of DRN-ChrimsonR projecting axons in the mPFC (50 ms at 5 Hz, 550 nm) induced transient EPSC depression in mPFC neurons (0.83 ± 0.02, *n* = 7 cells, *n* = 4 mice; Paired *t* test, *P* < 0.001) (Extended Data Fig. 4f, g). In contrast, neuronal recordings from Cort-mice showed an enhanced and persistent depression of synaptic transmission for at least 30 min after 5-HT local stimulation (0.42 ± 0.06, *n* = 9 cells, *n* = 5 mice; Paired *t* test, P < 0.001) (Fig. 3b, c).

The presence of 5-HT can affect miniature excitatory synaptic responses (mEPSCs) decreasing the frequency of synaptic events [113]. In line with those studies, naïve animals showed a significant decrease of the mEPSCs frequency after 5-HT stimulation (0.72 ± 0.06, *n* = 8 cells, *n* = 2 mice, One Way ANOVA, Tukey Test, *P* = 0.012), without affecting the amplitude (0.95 ± 0.04, One Way ANOVA, Tukey Test, *P* = 0.105) (Extended Data Fig. 2c, d). However, in Cort-mice neither frequency (1.30 ± 0.33, *n* = 11 cells, *n* = 3 mice, One Way ANOVA, Tukey Test, *P* = 0.247) nor amplitude after 5-HT stimulation (1.02 ± 0.02, One Way ANOVA, Tukey Test, *P* = 0.700) (Extended Data Fig. 2c, d) were affected, confirming that 5-HT-driven synaptic plasticity was altered in depressive-like states.

### 5-HT-induced synaptic plasticity requires astrocyte glutamatergic signaling

By regulating structural and physiological features of the synapses, astrocytes have been shown to play critical roles controlling synaptic transmission and plasticity [114]. Hence, we evaluated whether astrocytes might contribute to 5-HT-driven synaptic plasticity. First, Ca^2+^ signaling was impaired by dialyzing the Ca^2+^ chelator BAPTA (20 mM) into the astrocyte syncytium through the recording pipette [70] in naïve mice, and 30 min later EPSCs at mPFC neurons were recorded (Fig. 3d). In these conditions, 5-HT local stimulation induced a long-lasting depression of EPSC amplitude (0.67 ± 0.07, *n* = 6 cells, *n* = 5 mice; Paired *t* test, *P* = 0,004) (Fig. 3e–g), similar to 5-HT-evoked responses found in Cort-mice (Fig. 3b, c). Accordingly, neuronal recordings from *Ip3r2*^*–/–*^ mice showed a remarkably sustained depression of EPSC amplitude after 5-HT stimulation (0.69 ± 0.09, *n* = 13, *n* = 10 mice; Paired *t* test, *P* = 0,006) (Fig. 3e–g), supporting the critical role of astrocytic Ca^2+^ signaling for the 5-HT-mediated synaptic plasticity in cortical circuits.

**Fig. 4:**
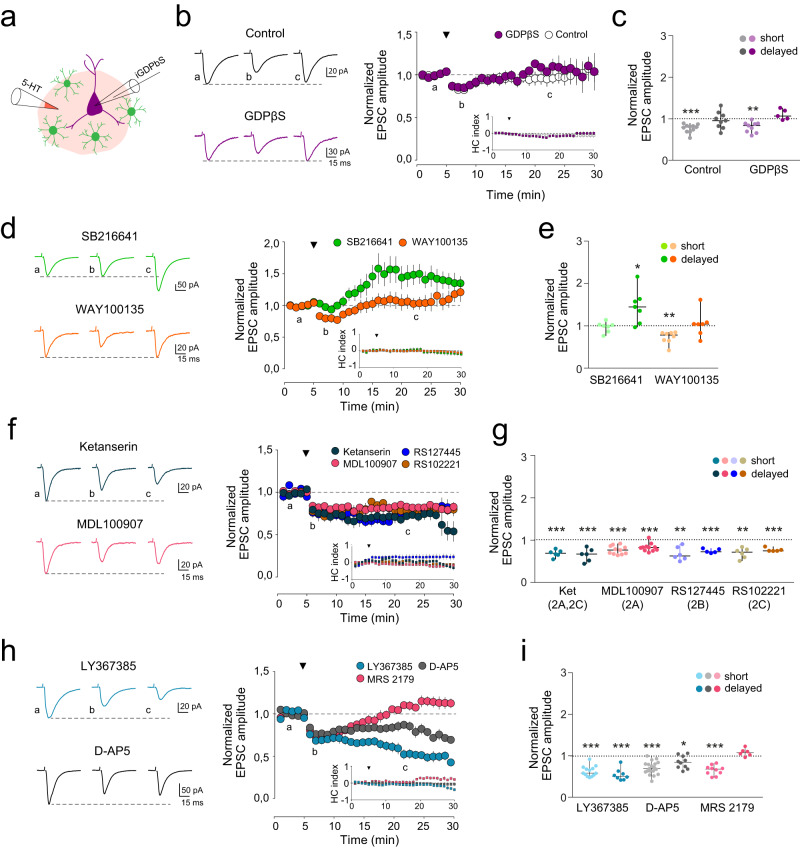
Serotonergic and glutamatergic receptors contribute to 5-HT-evoked synaptic plasticity. **a** Scheme of intracellular loading of GDPβS in recording neuron and 5-HT local application in the same field. **b** Left, representative EPSC average traces (average from 20 consecutive responses) recorded from pyramidal neurons before (**a**), and after (**b**, **c**) 5-HT application in control mice (black) and in GDPβS filled neurons-control mice (purple). Short (**b**) and delayed (**c**) synaptic responses are shown. Right, average of normalized EPSC amplitude and Holding Current (HC) index over time before and after 5-HT application in control (*n* = 11 cells, *n* = 7 mice) and GDPβS-postsynaptic mice (*n* = 9 cells, *n* = 5 mice). Black triangle denotes 5-HT puff application for the entire figure. Data shown as mean ± s.e.m. **c** Scatter plot of EPSC amplitude changes recorded during the first 5 min for short, and after 25 min for delayed synaptic effects; Paired *t* test, *P* ≤ 0.001. After intracellular loading of neurons with GDPβS, 5-HT induced the same response as in control condition (One Way ANOVA, *P* > 0.05). Data shown as median ± range (min and max values). **d** Left, representative EPSC average traces (average from 20 consecutive responses) recorded from pyramidal neurons before (**a**), and after (**b**, **c**) 5-HT application in presence of SB216641 (green) and WAY100135 (orange) in control mice. Right, average of normalized EPSC amplitude and HC index over time before and after 5-HT application in presence of SB216641 (*n* = 7 cells, *n* = 4 mice) and WAY100135 (*n* = 8 cells, *n* = 4 mice) in control mice. **e** Scatter plot of EPSC amplitude changes recorded during the first 5 min for short, and after 25 min for delayed synaptic effects. 5-HT1BR blockade induce a persistent synaptic potentiation of excitatory synaptic transmission (Paired *t* test, *P* = 0.023). **f** Left, representative EPSC average traces (average from 20 consecutive responses) recorded from pyramidal neurons before (**a**), and after (**b**, **c**) 5-HT application in presence of ketanserin (blue) and MDL100907 (pink) in control mice. Right, average of normalized EPSC amplitude, and HC index over time before and after 5-HT application in presence of ketanserin (*n* = 6 cells, *n* = 3 mice), MDL100907 (*n* = 11 cells, *n* = 4 mice), RS127445 (*n* = 6 cells, *n* = 2 mice), and RS102221 (*n* = 6 cells, *n* = 2 mice) in control mice. **g** Scatter plot of EPSC amplitude changes recorded during the first 5 min for short, and after 25 min for delayed synaptic effects. 5-HT2R family antagonist induced a persistent synaptic depression of excitatory synaptic transmission (Paired *t* test, *P* = 0.002 for ketanserin, *P* < 0.001 for MDL100907, RS127445 and RS102221). **h** Left, representative EPSC average traces (average from 20 consecutive responses) recorded from pyramidal neurons before (**a**), and after (**b**, **c**) 5-HT application in presence of LY367385 (blue) and D-AP5 (gray) in control mice. Right, average of normalized EPSC amplitude and HC index over time before and after 5-HT application in presence of LY367385 (*n* = 13 cells, *n* = 6 mice), D-AP5 (*n* = 18 cells, *n* = 7 mice) and MRS 2179 (*n* = 10 cells, *n* = 5 mice) in control mice. **i** Scatter plot of EPSC amplitude changes recorded during the first 5 min for short, and after 25 min for delayed synaptic effects. LY367385 and D-AP5 induced a persistent synaptic depression of excitatory synaptic transmission (Paired *t* test, *P* = 0.008 for LY367385, *P* = 0.010 for AP5 *P < 0.05, **P < 0.01, ***P < 0.001).

We further investigated the contribution of the postsynaptic 5-HT receptors to the observed responses by including GDPβS, a selective blocker of G-protein activity, into the recording pipette (Fig. 4a). In these conditions, where all postsynaptic receptors coupled to G-protein signaling were blocked, including metabotropic 5-HTRs, 5-HT stimulation induced similar transient EPSC depression to control recordings (0.80 ± 0.04 in GDPβS, *n* = 9 cells, *n* = 5 mice vs 0.77 ± 0.03 in control, *n* = 11 cells, *n* = 7 mice; One Way ANOVA, *P* = 0.572) (Fig. 4b, c), indicating that postsynaptic metabotropic 5-HT receptors did not contribute to the 5-HT driven synaptic plasticity. Presynaptic 5-HT receptors have been found to induce strong modulation of synaptic transmission [115], with particular attention to 5-HT1A and 5-HT1B, which are located on both serotonergic and non-serotonergic presynaptic terminals throughout the brain and induce strong inhibition of neurotransmitter release [112, 116]. Therefore, we next investigated the role of 5-HT1B and 5-HT1A receptors in the synaptic depression evoked by 5-HT in mPFC. The presence of SB216641 (50 µM), a selective antagonist of 5-HT1B, did prevent the EPSC depression induced by 5-HT stimulation (0.97 ± 0.05, *n* = 7 cells, *n* = 4 mice; Paired *t* test, P = 0.557) (Fig. 4d, e). Indeed, a sustained EPSC potentiation was found in the presence of 5-HT1B blocker (1.46 ± 0.15; Paired *t* test, *P* = 0.023) (Fig. 4d, e). In contrast, the blockade of 5-HT1A with WAY100135 (10 μM) did not prevent the synaptic depression induced by 5-HT (0.73 ± 0.05, *n* = 8 cells, n = 4 mice; Paired *t* test, *P* = 0.001) (Fig. 4d, e), suggesting a predominant role of 5-HT1B receptors for the 5-HT-driven synaptic inhibition accounting in mPFC layer 2/3 [112]. The role of 5-HT1D to the 5-HT-mediated effects has not been tested in our study, so its potential contribution cannot be ruled out. According to the 5-HT-driven astrocyte Ca^2+^ signaling and 5-HT2Rs signaling, we next investigated whether 5-HT2Rs, 5-HT2A, 5-HT2B and 5-HT2C, were involved in the reported synaptic plasticity. The blockade of 5-HT2A receptor with ketanserin (10 μM) or MDL100907 (1 μM) did induce long-lasting EPSC depression (Fig. 4f, g), with similar results obtained by perfusing selective antagonists of 5-HT2B and 5-HT2C receptors (Fig. 4f, g). Remarkably, this sustained synaptic depression simulated that observed by downregulating Ca^2+^ signaling in astrocytes (Fig. 3e–g). All in all, present data suggest the cooperative actions of presynaptic 5-HT1B and astrocytic 5-HT2 receptors would account for the net effect of 5-HT-mediated synaptic plasticity.

By releasing active substances, such as glutamate, D-serine, ATP, among others, astrocytes modulate synaptic plasticity and behavior [7]. We next studied the role of these transmitters in 5-HT-driven synaptic plasticity. The presence of LY367385 (50 μM), a selective antagonist for metabotropic glutamate receptors type 1a (mGluR1a), and AP5 (50 μM), the NMDA receptor antagonist, evoked a long-lasting EPSC depression after 5-HT stimulation (0.54 ± 0.09 and 0.84 ± 0.05, respectively; n ≥ 5 cells, *n* ≥ 3 mice; Paired *t* test, *P* ≤ 0.010) (Fig. 4h, i). MRS 2179 (10 μM), a selective antagonist of purinergic receptors type P2Y1, did not alter the 5-HT-mediated synaptic responses (1.08 ± 0.04, *n* = 6 cells, *n* = 2 mice; Paired *t* test, *P* = 0.086) (Fig. 4h, i). Overall, these results suggest that 5-HT engaged astrocytic glutamate release through 5-HT2R activation, which further activated neuronal mGluR1 and NMDA receptors that contributed to the 5-HT-driven synaptic plasticity in mPFC (Extended Data Fig. 7j). In contrast, Cort-mice showed a reduced 5-HT-mediated astrocytic Ca^2+^ signaling, that failed to stimulate the release of glutamate (Fig. 2j, k), and correlated with an aberrant 5-HT mediated synaptic plasticity.

### Boosting astrocyte Ca^2+^ signaling diminishes behavioral deficits in corticosterone-treated mice

**Fig. 5:**
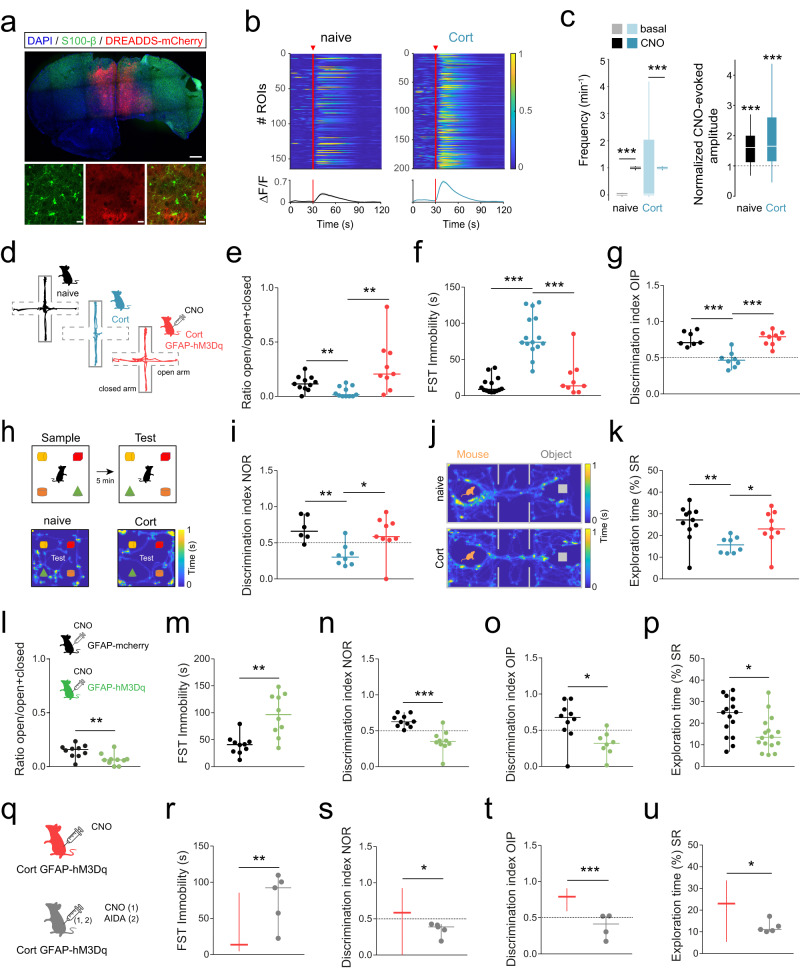
Boosted astrocytic Ca^2+^ signaling in mPFC ameliorates the behavioral deficits shown by Cort-mice. **a** Confocal imaging of immunohistochemistry-confirmed hM3Dq-mCherry expression (red) in astrocytes (S100-β, green). Scale bar, 500 μm (up), 50 μm (down). **b** Heatmaps of CNO-hM3D(Gq) evoked ROIs activity and average population activity in astrocytes of naïve (*n* = 164, *n* = 3 mice) and Cort-mice (*n* = 200, *n* = 2 mice). Red triangle and bar denote CNO puff application (1 mM, 2 s, 1 bar). Data are presented as mean ± s.e.m. Color code denotes fluorescence change. **c** Box and whisker (BW) plots representing fluctuations of Ca^2+^ astrocytic events induced by CNO in naïve and Cort-mice. Both mice showed an increased in normalized CNO-evoked amplitude, Paired *t* test, *P* < 0.001. **d** Scheme and representative activity traces in EPM for naïve, Cort-mice and Cort-GFAP-hM3Dq mice stimulated with CNO (3 mg/kg, i.p.). **e** EPM exploration index was enhanced in Cort-GFAP-hM3Dq mice vs Cort-mice, showing more entries into the open arms. One Way ANOVA, Dunn’s method, *P* = 0.003. Data are presented as median ± range for the entire figure. **f** FST immobility time was rescued in Cort-GFAP-hM3Dq mice (One Way ANOVA, Tukey test, *P* < 0.001), reaching similar values to naïve mice (One Way ANOVA, *P* = 0.413). **g** OIP discrimination index reduced in Cort-mice (*P* < 0.001) was increased after CNO administration. One Way ANOVA, Tukey test, *P* < 0.001. **h** OIP test scheme and representative spatial heatmaps of naïve and Cort-mice performing the task. Color code denotes accumulated time. **i** NOR discrimination index reduced in Cort-mice (One Way ANOVA, Tukey test, *P* = 0.001) was rescued after CNO hM3Dq-astrocyte stimulation (One Way ANOVA, Holm-Sidak method, *P* = 0.021). **j** Representative spatial heatmaps of naïve and Cort-mice during SR task. Color code denotes accumulated time. **k** Exploration time analyzed for naïve, Cort-mice and Cort-GFAP-hM3Dq mice during SR test showing that reduced levels of social interaction found in Cort-mice (One Way ANOVA, Holm-Sidak method, *P* = 0.004) were reverted by CNO administration (One Way ANOVA, Dunn´s method, *P* = 0.030). **l** EPM exploration index in naïve GFAP-hM3Dq mice was reduced compared with control GFAP-mcherry naïve mice after CNO administration (3 mg/kg, i.p.). One Way ANOVA, Holm-Sidak, *P* = 0.007. **m** FST immobility time showed enhanced values in GFAP-hM3Dq mice. One Way ANOVA, Tukey Test, *P* = 0.002. **n**, **o**, **p** NOR discrimination index, OIP discrimination index and SR exploration time were reduced in GFAP-hM3Dq mice. One Way ANOVA, Dunn’s Method, *P* < 0.001 for NOR; Tukey Test, *P* = 0.011 for OIP; Tukey Test, *P* = 0,015 for SR. **q** Scheme of CNO and AIDA (5 mg/kg) i.p. administration in Cort-GFAP-hM3Dq mice. 1 and 2 denotes sequential i.p. injections. **r** FST immobility time showing increased values for Cort-GFAP-hM3Dq+AIDA mice (gray) compared with Cort-GFAP-hM3Dq mice (red, shown as median ± range as reference). One Way ANOVA, Tukey test *P* = 0,008. **s**, **t**, **u** NOR discrimination index, OIP discrimination index and SR exploration time were reduced by previous administration of AIDA in Cort-GFAP-hM3Dq mice. One Way ANOVA, Dunn’s Method, *P* = 0,020 for NOR; Tukey test, *P* < =0.001 for OIP; Dunn’s Method, *P* = 0,015 for SR. The center line in plots indicates the median, the top and bottom edges indicate the 25th and 75th percentiles, respectively, and the whiskers extend to the maximum and minimum data points. *P < 0.05, **P < 0.01, ***P < 0.001.

In addition to the mood and affective responses, MDD also impairs cognitive abilities associated with attention, executive functions, learning and memory related processes [24, 117]. Alterations in astrocyte Ca^2+^ signaling and gliotransmission have been shown to impact executive functions, such as fear-related behaviors, decision-making, and working memory tasks [118]. Hence, we investigated whether promoting the intracellular Ca^2+^ in astrocytes might have significant impact on behavioral performance in Cort-mice. First, astrocytes from mPFC were activated by the selective expression of the designer receptors exclusively activated by designer drugs (DREADDs; AAV5-GFAP-hm3D(Gq)-mCherry) (Fig. 5a). The presence of the selective agonist clozapine-N-oxide (CNO, 1 mM) induced robust Ca^2+^ responses in transfected astrocytes from both naïve and Cort-mice (Fig. 5b, c), confirming their ability to engage Ca^2+^ signaling in mPFC astrocytes. Next, we performed behavioral test on Cort-mice previously injected with AAV5-GFAP-hm3D(Gq)-mCherry in mPFC, and scores after 20 min of CNO injection (3 mg/kg i.p) were analyzed (Fig. 5d–k; Extended Data Fig. 7a). Remarkably, selective activation of mPFC astrocytes by Gq-DREADDS ameliorated the altered rates of animal despair (from 83.68 ± 7.44 s in Cort-mice to 24.33 ± 8.45 s in Cort-GFAP-DREADDs mice, *n* = 15 vs *n* = 9, respectively; One Way ANOVA, Tukey test; *P* < 0.001) and anxiety levels found in Cort-mice (from 0.04 ± 0.01 to 0.28 ± 0.08; One Way ANOVA, Dunn´s method; *P* = 0.003) (Fig. 5d–f). Additionally, the cognitive abilities impaired by the Cort-treatment were significantly improved after astrocytic Gq-DREADDS stimulation (from 0.48 ± 0.04 to 0.77 ± 0.03, One Way ANOVA, Tukey test, *P* < 0.001, in OIP) (Fig. 5g–i) reaching similar values to those shown by naïve mice. Moreover, social interactions were also reestablished by selective activation of mPFC astrocytes in Cort-mice (from 15.67 ± 1.32% to 23.45 ± 2.80% exploration time; One Way ANOVA, Dunn´s method; *P* = 0.030) (Fig. 5j, k). Therefore, the manipulation of astrocyte Ca^2+^ signaling in mPFC is able to counteract the depressive-like behaviors shown by Cort-mice.

In contrast, CNO stimulation of mPFC astrocytes expressing Gq-DREADDS negatively affected mouse performance in naïve mice. Indeed, after CNO administration, naïve mice showed altered values in the behavioral tests, worsening the animal despair levels (from 40.12 ± 5.63 to 96.36 ± 12.27 s; *n* = 10; One Way ANOVA, Tukey test *P* = 0.002), anxiety levels (from 0.14 ± 0.02 in naïve mice to 0.07 ± 0.02 in naïve-DREADDS mice; One Way ANOVA, Holm-Sidak test; *P* = 0.007), as well as cognitive (from 0.64 ± 0.03 to 0.35 ± 0.05 in NOR; One Way ANOVA, Tukey test; *P* < 0.001), and social interaction abilities (from 21.60 ± 2.54 % to 14.91 ± 2.01%; One Way ANOVA, Tukey test; *P* = 0.015) (Fig. 5l–p). Notably, these alterations were similar to those found by Cort-treatment. Control experiments using CNO in AAV5-GFAP-mCherry transfected mice showed no significant side effects [119], with similar cognitive performance to those naïve mice treated with saline or without viral injection manipulations (Extended Data Fig 7b–d). Additionally, the synaptic responses induced by Gq-DREADDS in astrocytes were evaluated in mPFC slices. CNO-driven astrocyte Ca^2+^ signals induced potentiation of EPSC amplitude in both naïve and Cort-mice (1.62 ± 0.22 in naïve and 1.39 ± 0.14 in Cort-mice, *n* ≥ 6 cells and *n* ≥ 3 mice; Paired *t* test; *P* < 0.05) (Extended Data Fig. 7e–g). In line with 5-HT driven astrocyte activation (Fig. 4h), EPSC potentiation induced by Gq-DREADDs astrocytes was impaired by selective blockade of mGluR1 with LY367385 (50 µM) (0.98 ± 0.06 in naïve and 0.94 ± 0.13 in Cort-mice, *n* ≥ 6 cells and *n* ≥ 3 mice; Paired *t* test; *P* > 0.05) (Extended Data Fig. 7e–g). CNO also enhanced the frequency of the NMDA-mediated currents, SICs, supporting that ability of astrocytes to release glutamate that impacts neuronal membranes, and showing that the capability of astrocytes to release glutamate was not compromised by Cort-treatment.

According to previous data, a critical role of mGluR1 for executive functions has been described [70, 120]. Consistently, we found that the systemic administration of AIDA (5 mg kg^−1^ i.p.), a selective antagonist of mGluR1, in naïve mice had a negative impact on behavior (Extended Data Fig. 8a–e). Remarkably, AIDA administration blocked the improved behavioral responses in Cort-mice after Gq-astrocytic stimulation with CNO (Fig. 5q–u), supporting the close relationship between astrocyte glutamatergic signaling and cognitive abilities [70]. In addition, the altered animal performance shown by naïve mice with astrocyte-expressing DREADDS after CNO stimulation was not modified by the presence of AIDA (Extended Data Fig. 8f–i). Overall, these data expose the highly regulated connection between astrocyte Ca^2+^ signals and animal behavior.

## Discussion

This study shows for the first time the abnormal neuron-astrocyte signaling in a mouse model that recapitulates depressive-like states, linking dysfunctional astrocyte Ca^2+^ dynamics and 5-HT-driven synaptic plasticity with behavioral impairments. According to previous studies [33, 55], we found that Cort-treatment in juvenile mice induced significant alterations in animal behavior mimicking some cognitive and mood features of MDD. Indeed, in vivo recordings in Cort-mice revealed an exacerbated astrocyte Ca^2+^ activity in mPFC with increased frequency and magnitude of Ca^2+^ events during open field exploratory activity. Remarkably, during social interactions, when mPFC activity is engaged [121, 122], astrocyte Ca^2+^ signaling was largely impaired in Cort-mice, in line with weakened animal performance. However, the amplitude of astrocyte Ca^2+^ events during non-animated object explorations was similar in naïve and Cort-mice (Fig. 1g, h), according to comparable rates between groups for object explorations. These data uncover the specific engagement of mPFC astrocytic networks during social behaviors, and allow to hypothesize that the value of the explorations might have a significant impact in cortical astrocytes, which would require further investigation.

The serotonergic system plays a central role in the pathophysiology and treatment of depression [90]. Despite of the controversy [34], there is evidence supporting the link between low levels of 5-HT and MDD [103, 104]. Here, we confirmed the aberrant serotonergic neurotransmission in Cort-mice by using iSeroSnFR, a serotonergic sensor expressed in mPFC astrocytes. We have observed reduced levels of 5-HT released by DRN projections to mPFC in Cort-mice, which showed diminished astrocytic Ca^2+^ events amplitude, as well as gliotransmission impairments after 5-HT stimulation in mPFC. In fact, although Cort-mice showed enhanced gliotransmission in resting conditions, with an exacerbated glutamate release by astrocytes, we found reduced 5-HT engaged gliotransmission in these mice. These results suggest that alterations in astrocyte Ca^2+^ signaling induced by depressive-like states have also significant impact on astrocyte-to-neuron signaling [123]. Nevertheless, alterations in Ca^2+^ dynamics shown by Cort-astrocytes were not restricted to 5-HT-driven signaling. For instance, abnormal Ca^2+^ responsiveness was also found after ATP stimulation, indicating that dysfunctional astrocytic Ca^2+^ in depressive-like states may impact a broad neuron-astrocyte and glial-glial signaling pathways. It is important to note that giving the different experimental approaches and conditions to record astrocyte Ca^2+^ signals from in vivo (Fig. 1) and ex vivo GCaMP6f transfected astrocytes (Fig. 2), with neuronal activity reduced in the presence of TTX, the obtained results have to be interpreted independently.

5-HT modulation of synaptic transmission and plasticity has been previously described as enhancing or reducing excitatory and inhibitory synaptic activity through the activation of different 5-HT receptor subtypes (5-HTRs) [108]. We found that 5-HT induced synaptic plasticity in layer 2/3 of mPFC principal neurons by leading a transient depression of EPSCs. Such 5-HT-mediated plasticity required the activation of 5-HT1B receptors, which localize predominantly in axon terminals regulating neurotransmitter release [112] and also astrocytic Ca^2+^-dependent signaling. Indeed, reducing astrocytic Ca^2+^ signaling by intracellular BAPTA-loading or by using *Ip3r2*^*–/–*^ mice, 5-HT-driven synaptic plasticity was impaired and a sustained depression of EPSCs was found. Similar effects were observed by blocking 5-HT2Rs family, which also mediated the 5-HT-driven Ca^2+^ signaling in astrocytes. Additionally, selective blockade of both mGluR1Rs and NMDARs resulted in long-lasting depression of 5-HT-induced synaptic plasticity. Overall, these results suggest a significant contribution of both astrocyte Ca^2+^ signaling and astrocytic glutamatergic signaling for serotonergic synaptic actions.

5-HT2Rs family, including 5-HT2A, 5-HT2B and 5-HT2C, are highly expressed in different neuronal cell types, but also astrocytes [91]. Several evidences have related 5-HT2Rs with pathophysiology of MDD [124], 5-HT2A and 5-HT2C in mPFC play a critical role in the regulation of mood disorders, and their antagonism has been related to antidepressant features [125]. Although our data support the activation of 5-HT2A located at the astrocytic membranes, the contribution of presynaptic 5-HT2A to the 5-HT-driven synaptic plasticity cannot be discarded [126, 127]. In that scenario, our data suggest that, in addition to neuronal partners, astrocytic 5-HT2 receptors might be a potential target for specific antagonists contributing to the antidepressant effects. Overall, present data suggest that presynaptic 5-HT1B and putative astrocytic 5-HT2 receptors contributes to the 5-HT-mediated synaptic plasticity, revealing synergies between neuronal and astrocyte serotonergic receptors for synaptic plasticity with relevant functional outcomes.

In addition to the astrocytic Ca^2+^ signaling alterations found in this study, other critical changes have been found in astrocytes in depressive-like states [46]. In this context, altered expression of glutamate transporter-1 (GLT-1) was described in MDD patients [128], but also selective blockade of GLT-1 induced depressive-like behaviors in rodents [18], both affecting glutamatergic signaling. Considering the intracortical regional differences in behavioral responses found between infralimbic and prelimbic mPFC areas after GLT-1 blockade [18], further studies are required to uncover whether the astrocyte Ca^2+^ signaling at specific intracortical structures has particular impact for depressive-like states.

Remarkably, hM3Dq-boosted astrocyte signaling in mPFC also impacts both intracortical connectivity and long-range projections from mPFC to subcortical brain regions [129], including hippocampus and amygdala, key brain areas involved in the spatial and memory tasks, and in anxiety-related behaviors, respectively. Here, we studied the role of astrocytic signaling in spatial exploration and novelty preference, as well as in mood behavior. We found that boosted astrocytic Ca^2+^ in prefrontal cortex overcomes depressive-like behavior. Indeed, selective hM3Dq activation of mPFC astrocytes in Cort-mice restored the behavioral scores to the naïve control values, including the social impairments. Downregulation of astrocytic Ca^2+^ signaling, based on transgenic *Ip3r2*^*–/–*^ mice [130, 131], has been related with depressive-like behaviors; our data support these findings and provide the first evidence showing the restoring of cognitive abilities in Cort-mice by targeting astrocytic Ca^2+^ in mPFC. In line with ex vivo data, selective blockade of mGluR1 with AIDA injections occluded the beneficial actions of CNO for behavioral performance in Cort-mice, supporting the role of mGluR1 in cognitive functions [70, 120] and suggesting that selective glutamatergic signaling from astrocytes may inspire the observed effects (Extended Data Fig. 7j). The FST is frequently used to investigate depressive-like behaviors; however, immobility can also be interpreted as a passive coping strategy nowadays [132]. Regardless the interpretation, here we observed that selective CNO astrocyte Ca^2+^ stimulation in mPFC induced changes in swimming strategies for both naïve and Cort-mice, shifting to higher immobility rates or restoring control values, respectively; which highlights the potential of mPFC astrocytes to modulate coping strategies.

The chronic corticosterone treatment approach used here has been found to recapitulate most of the endophenotypes related to depression based on the current Research Domain Criteria (RDoC) system [133], that is, avoidance of negative valence behaviors [33, 55], detachment of positive valence behaviors [30, 55] and impaired social and cognitive abilities [134–137], revealing it as a suitable experimental protocol to study behavioral consequences of chronic stress-related diseases. Different chronic stress protocols are also commonly used to study anxiety and depressive phenotypes in animal research, including unpredictable chronic mild stress protocol and social defeat [27, 28]. Whether astrocyte Ca^2+^ signaling dysfunctions is a common feature of stress-related disorders or can be induced by different stress-related protocols needs to be resolved and addressed by future studies.

It has been reported that CNO is degraded into clozapine, a potent antipsychotic drug, which can potentially induce side effects [119]. Therefore, control experiments were performed in naïve expressing GFAP-mCherry viral vectors with CNO systemic application. These mice did not show significant behavioral changes in the analyzed tests (Extended Data Fig. 7b–d), suggesting that the reported behavioral effects of CNO were indeed attributed to selective stimulation of DREADDS-expressing astrocytes and not to direct modulation of dopamine or serotonergic receptors [119]. However, it cannot be excluded that clozapine could also be binding different molecular targets that are not considered in this study [138]. It is worth to mention, that similar hM3Dq strategy but in naïve mice was found harmful for animal behavior. In line with reported alterations of functional connectivity in prefrontal networks by CNO in healthy subjects [129], our data show that an increased astrocytic Ca^2+^ signaling in mPFC disrupted neuronal activity leading to a detrimental performance in mood and cognitive-related tasks. It has been shown that in the hippocampus, hM3Dq activation of astrocytes enhanced memory abilities enhancing excitatory synaptic transmission [139, 140], supporting a positive role of enhancing astrocyte Ca^2+^ signaling for hippocampal-dependent contextual memory task [139]. In contrast, present data showed that activation of mPFC astrocytes, despite the potentiation of excitatory synaptic transmission (Extended Data Fig. 7e–g), failed to induce beneficial behavioral effects in control mice. In line with our results, experiments performed in control animals have shown that Gq-DREADDs in visual cortex lead to significant impairments in sleep-wake transitions, decreasing the percent time awake [141], and selective astrocyte activation in cingulate cortex triggers neuronal hyperactivity and increases BOLD functional connectivity between cortical and hippocampal regions, similar to the abnormal activity found in Alzheimer disease [129]. Then, these findings highlight the diversity of astrocyte-neuron circuits and propose that regional differences should be considered for the interpretation of behavioral outcomes when targeting astrocytes with Gq-DREADDs. It is important to note that present results were found in male mice, but sex differences have been described by functional studies in Cort-mice [31, 142–144]; therefore, an exhaustively comparative analysis between male and female mice will provide evidence of sex differences in Ca^2+^ dynamics and 5-HT-driven astrocyte-neuron signaling in stress-related depressive-like behaviors.

Glutamatergic dysfunction and NMDARs play a critical role in psychiatric disorders, including MDD [145]. Thus, direct targeting of the NMDA receptor with ketamine [146] results in faster antidepressant effects compared with classical antidepressants, including selective serotonin reuptake inhibitors, whose effects might take several weeks in MDD patients. Here, we showed that targeting astrocyte Ca^2+^ signaling in mPFC induced fast antidepressant effects in Cort-mice, which might be considered as a potential target for antidepressant drugs. As for the case of ketamine [147], the overactivation of astrocytic Ca^2+^ resulted in a significant increase of depressive symptoms in naïve mice. Thus, stimulating astrocyte Ca^2+^ signaling does not produce beneficial effects in healthy control subjects. These findings will need further exploration for understanding the mechanisms underlying hM3Dq-astrocyte driven dysfunctional cortical networks and animal behavior in healthy conditions. Our study was focused on the acute response to astrocyte activation, and longitudinal studies would be required to investigate long-lasting effects of CNO-stimulated astrocytes in Cort-mice.

Overall, the present study reveals the critical role of astrocytes and the tight control of their Ca^2+^ levels for the top-down regulation of executive functions driven by the mPFC in health and stress-induced depressive-like phenotypes [130]. Moreover, our data highlight the potential value of astrocytes as targets for developing improved treatments for these neurological disorders.

### Supplementary information

Supplementary Figures 1-8

Source Data Fig 1

Source Data Fig 2

Source Data Fig 3

Source Data Fig 4

Source Data Fig 5

Source Data Extended_Data Fig 1

Source Data Extended_Data Fig 2

Source Data Extended_Data Fig 3

Source Data Extended_Data Fig 4

Source Data Extended_Data Fig 5

Source Data Extended_Data Fig 6

Source Data Extended_Data Fig 7

Source Data Extended_Data Fig 8

## Data Availability

All data generated or analyzed during this study are either included in this published article or are available from the corresponding author upon reasonable request. Source data are provided with this paper.

## Code availability

Analyses used in this study are largely standard approaches for this type of data. The code that supports these findings is available upon request from the corresponding author.
