# Supplementary material for: Dysfunctional serotonergic neuron-astrocyte signaling in depressive-like states
Source: Mol Psychiatry. 2023 Sep 29;28(9):3856–73. doi: 10.1038/s41380-023-02269-8 (PMC10730416; doi:10.1038/s41380-023-02269-8)
Supplement: Supplementary file 1 — Supplementary Figures 1-8 [file 41380_2023_2269_MOESM1_ESM.pdf]

Dysfunctional serotonergic neuron-astrocyte signaling in depressive-like states

Candela González-Arias[1], Andrea Sánchez-Ruiz[1], Julio Esparza[1], Cristina Sánchez-Puelles[1,2], Lucia Arancibia[1], Jorge Ramírez-Franco[3], Davide Gobbo[4], Frank Kirchhoff[4], Gertrudis Perea[1]

Supplemental Information

Extended Data Figures 1-8

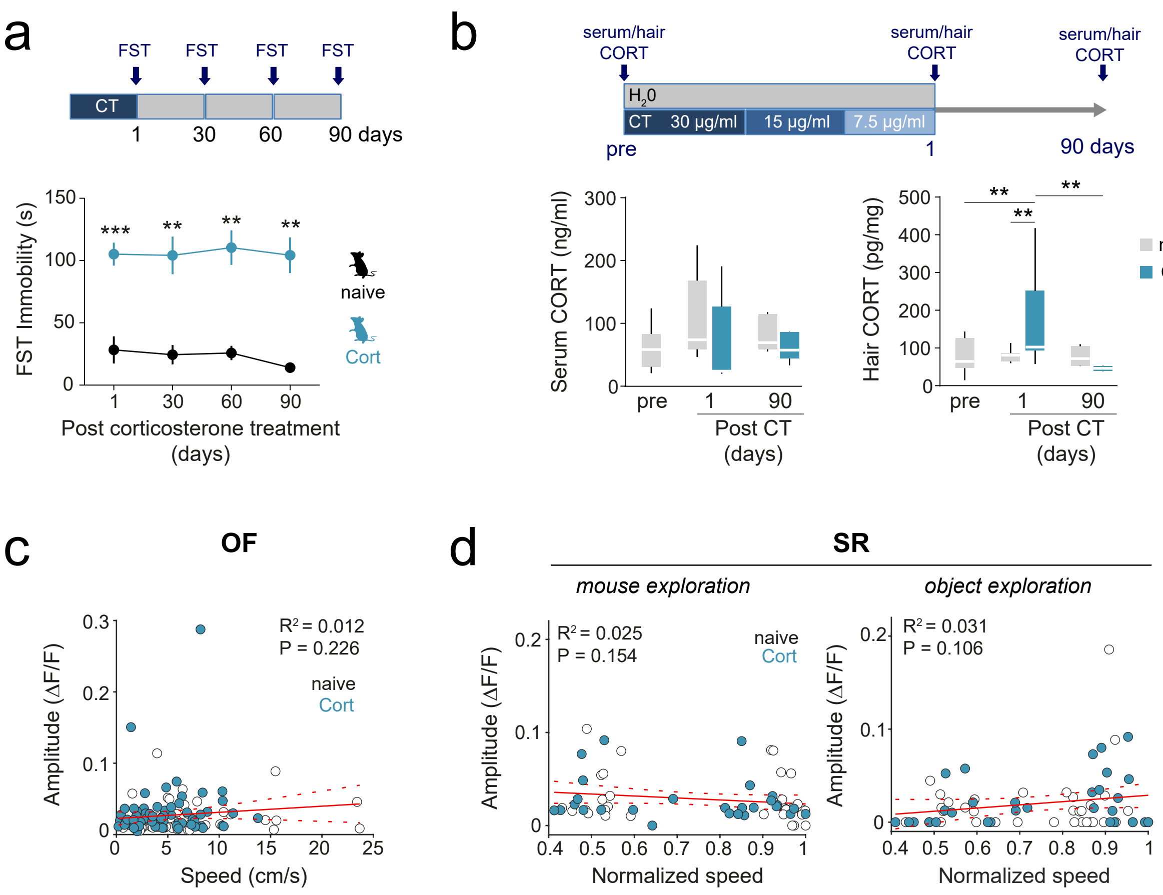

Extended Data Figure 1. Long-term effects of Cort-treatment and relationship between astrocytic Ca^{2+} responses and mouse movement. a) Long-term effects of corticosterone treatment (CT) analysed by FST. Note that Cort-mice showed increase immobility rates up to three months after treatment. One Way ANOVA, Holm-Sidak method/Dunn's method, P<0.001, 1 day after Cort treatment (n_{naive}=6, n_{Cort}=8); P=0.001 after 30 days (n_{naive}=6, n_{Cort}=8); P=0.002 after 60 days (n_{naive}=6, n_{Cort}=8); P=0.004 after 90 days (n_{naive}=3, n_{Cort}=3). Data are presented as mean ± s.e.m. **b)** *Top,* timeline of corticosterone measurements in hair and serum. *Bottom left*, serum corticosterone levels collected before (n=11 mice), 1 day (n_{naive}=10 mice, n_{Cort}=7 mice) and 90 days (n_{naive}=5 mice, n_{Cort}=5 mice) after CT. Note that there were no differences in serum corticosterone levels at different extraction points (Two Way ANOVA, P>0.05). *Bottom right,* hair corticosterone levels collected before (n=17 mice), 1 day (n_{naive}=10 mice, n_{Cort}=11 mice) and 90 days (n_{naive}=4 mice, n_{Cort}=4 mice) after treatment. Note that Cort-mice did show increased hair corticosterone levels 1 day after treatment (Two Way ANOVA, P=0,001) that recovered to control values 90 days after treatment (Two Way ANOVA, P=0,310). The center line in BW plots indicates the median, the top and bottom edges indicate the 25th and 75th percentiles, respectively, and the whiskers extend to the maximum and minimum data points. **c)** Relationship between the amplitude of Ca^{2+} activity and mean speed of mice during the open field (OF) test; n_{naive} = 56 trials, 5 naive mice, n_{Cort} = 64 trials, 3 Cort mice. **d)** Relationship between the amplitude of Ca^{2+} activity and the normalized speed of mice during the mouse and object exploration in a social recognition (SR) test. Object: n_{naive} = 35 trials, n_{Cort} = 30 trials. Mouse: n_{naive} = 34 trials, n_{Cort} = 27 trials; n= 7 naïve, 6 Cort mice. R^2: coefficient of determination, P: P-value for slope different from 0 derived from a linear regression model. Dashed lines show 95% confidence interval of the linear fit. **P<0.01, ***P<0.001.

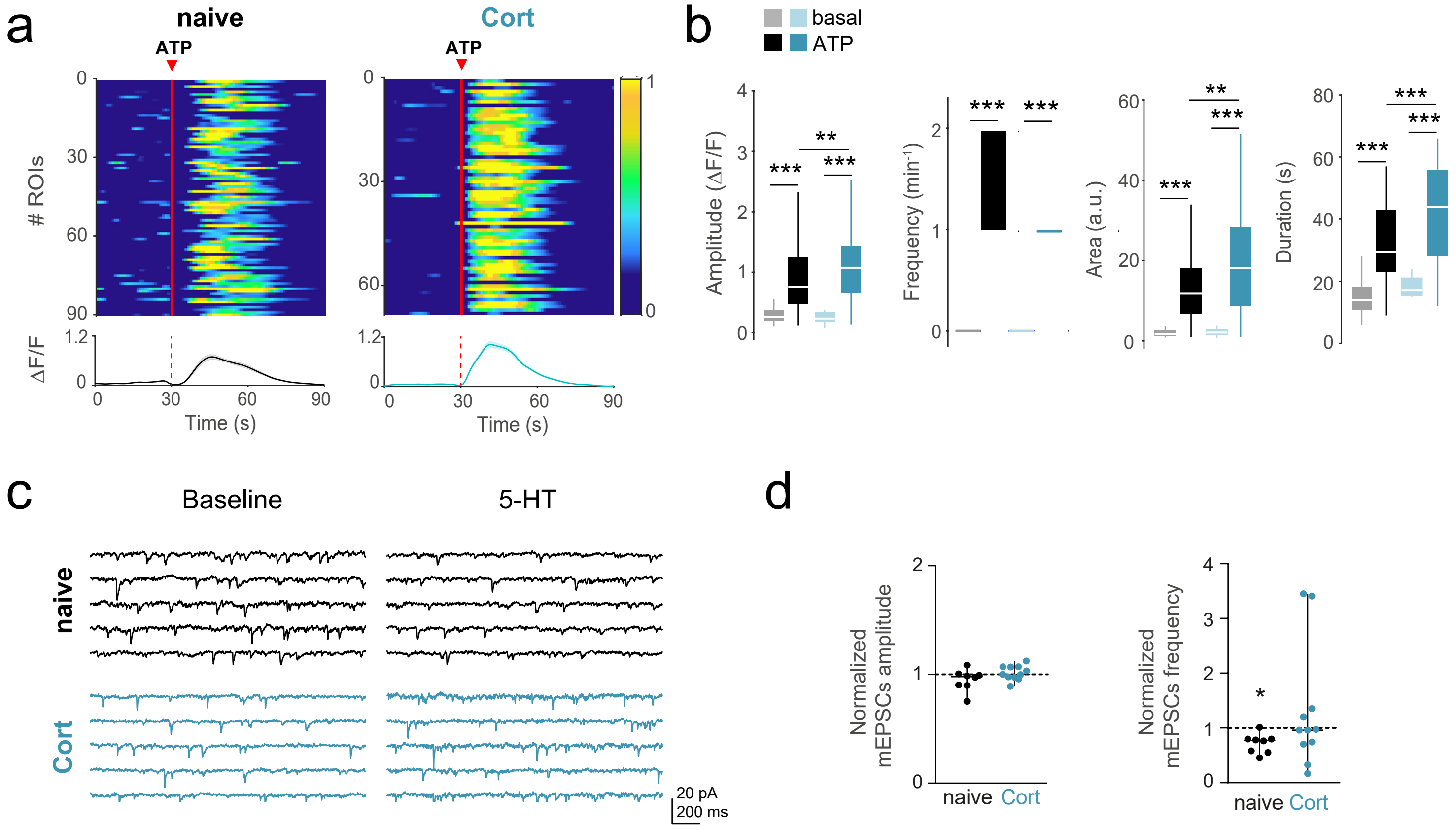

Extended Data Figure 2. Dysfunctional Ca^{2+} signaling and 5-HT-driven synaptic plasticity in Cort-mice. a) Heatmaps of ATP-evoked ROIs activity and average population activity in mPFC astrocytes loaded with Fluo-4 AM of naïve (n=90 ROIs, n=2 mice) and Cort-mice (n=68 ROIs, n=2 mice). Data are presented as mean ± s.e.m. Color code denotes fluorescence change. Red triangle and bar denote ATP puff application (1mM, 10s, 1 bar). **b)** Box and whisker (BW) plots representing the dynamics of Ca^{2+} astrocytic events in naïve and Cort-mice. ATP enhanced the amplitude, area and duration of Ca^{2+} events in Cort-mice compared to naïve mice. One Way ANOVA, Dunn's Method, P=0.006, P=0.002, P<0.001, respectively. The center line in BW plots indicates the median, the top and bottom edges indicate the 25th and 75th percentiles, respectively, and the whiskers extend to the maximum and minimum data points. **P<0.01, ***P<0.001. **c)** Representative traces of miniature EPSCs (mEPSC) before and after 5-HT in naïve and Cort-mice. **d)** Scatter plot of mEPSCs recorded before (5 min) and after 5-HT (5 min) showing a significant decrease in mEPSCs frequency in naïve mice (n=8 cells, n=2 mice), which was not present in Cort-mice (n=11 cells, n=3 mice). One Way ANOVA, Tukey test, P=0.012, 0.247, respectively. Data are presented as median ± range (min and max values). *P<0.05.

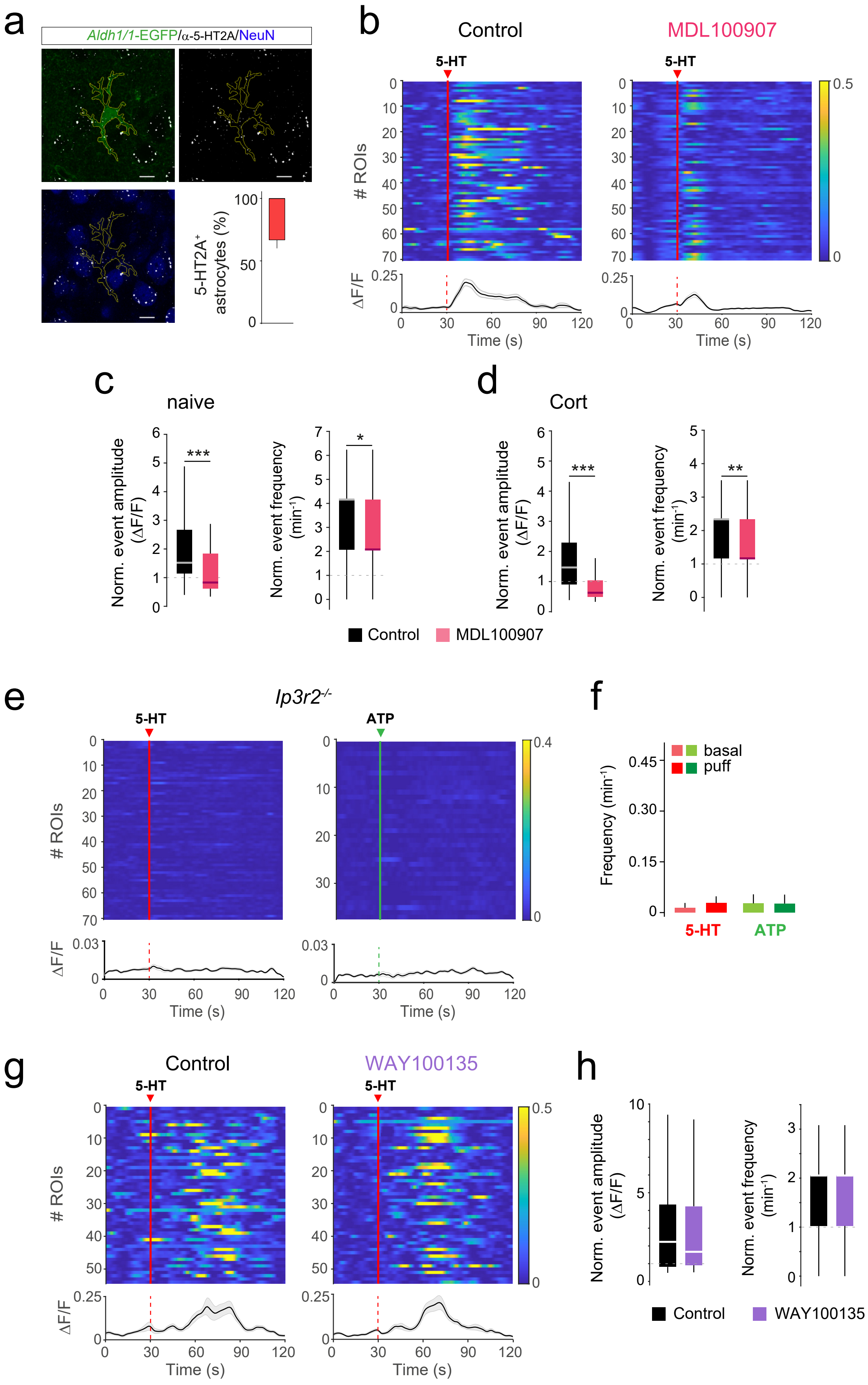

a
Aldh1/1-EGFP/α-5-HT2A/NeuN
5-HT2A+ astrocytes (%)
b
Control
MDL100907
5-HT
ROIs
ΔF/F
Time (s)
c
naive
Norm. event amplitude (ΔF/F)
Norm. event frequency (min-1)
***
*
d
Cort
***
**
Control
MDL100907
e
Ip3r2-/-
5-HT
ATP
ROIs
ΔF/F
Time (s)
f
Frequency (min-1)
basal
puff
5-HT
ATP
g
Control
WAY100135
5-HT
ROIs
ΔF/F
Time (s)
h
Norm. event amplitude (ΔF/F)
Norm. event frequency (min-1)
Control
WAY100135

Extended Data Figure 3. Astrocytic expression of 5-HT2AR and Ip3-dependent pathway for 5HT-driven Ca^{2+} responses. a) Representative confocal image showing an *Aldh1/1*-EGFP astrocyte (green), 5-HT2AR puncta (white) and the neuronal marker NeuN (blue). Scale bar 10 µm. Average percentage of 5-HT2AR+ astrocytes (n= 83 astrocytes, 3 slices, 2 mice). **b)** Heatmaps of 5-HT evoked ROIs activity and average population activity in astrocytes of control mice, in basal conditions (left), and in presence of 5-HT2A antagonist (MDL100907, right, n=70, n=2 mice). Data are presented as mean ± s.e.m. Color code denotes fluorescence change. Red triangle and bar denote 5-HT puff application (1mM, 10s, 1 bar). **c-d)** Box and whisker plots representing changes in amplitude and frequency of Ca^{2+} events induced by local 5-HT stimulation. Values were normalized to spontaneous activity before stimulation, in control (black) and after bath application of MDL100907 (pink) in naïve and Cort mice (n=70, n=3 mice). Note the reduced amplitude and frequency under the influence of MDL100907 for both control and Cort mice. Paired t-test, P<0.05. **e)** Heatmaps ROIs activity and average population induced by 5-HT (left) and ATP (right) in astrocytes loaded with Fluo-4 AM from *Ip3r2*$^{-/-}$ mice (n= 70 ROIs and n=37 ROIs, respectively; n=2 mice). Color code denotes fluorescence change. Red (5-HT) and green (ATP) triangles denote puff application (1 mM, 10s, 1 bar). **f)** Bar graph representing the frequency of astrocytic Ca^{2+} events before (basal) and after puff application. Note the absence of significant Ca^{2+} events.Paired t-test, P=1.000 (5-HT), P=1.000 (ATP). Data are presented as mean ± s.e.m. **g)** Heatmaps of 5-HT evoked ROIs activity and average population activity in basal conditions (left) and in presence of 5-HT1A antagonist (WAY100135, right, n=54, n=2 mice). Data are presented as mean ± s.e.m. Color code denotes florescence change. Red triangle and bar denote 5-HT puff application (1mM, 10s, 1 bar). **h)** Box and whisker plots representing changes in amplitude and frequency of Ca^{2+} events induced by local 5-HT stimulation. Values were normalized to spontaneous activity before stimulation, in control (black) and after bath application of WAY100135 (purple) in naïve mice. Note that in both conditions no differences in amplitude (Paired t-test, P= 0.145) and frequency (Paired t-test, P=0.079) were found after 5-HT application. The center line in BW plots indicates the median,the top and bottom edges indicate the 25th and 75th percentiles, respectively, and the whiskers extend to the maximum and minimum data points. *P<0.05, **P<0.01, ***P<0.001.

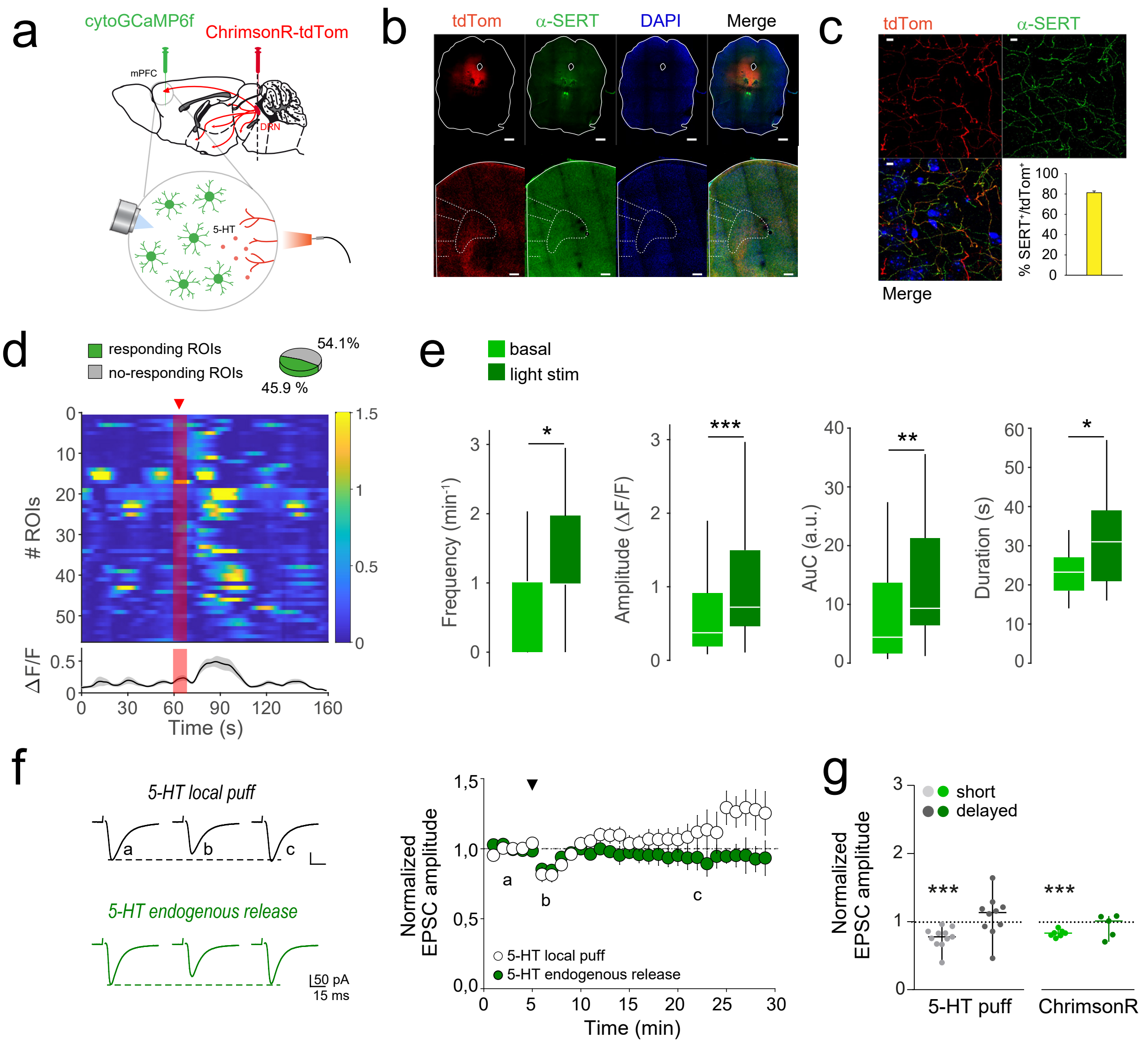

Extended Data Figure 4. Optogenetic stimulation of serotonergic afferences in mPFC. a) *Top*, schematic representation of viral injections in DRN with AAV9-hSyn-ChrimsonR-tdTom, and in mPFC with AAV5-GFAP-GCaMP6f in mPFC targeting astrocytes. Bottom, optogenetic stimulation DRN projections to mPFC and Ca^{2+} imaging recordings. **b)** Confocal imaging of immunohistochemistry showing AAV9-hSyn-ChrimsonR-tdTom (red), α-SERT (green) and DAPI nuclei (blue) localization in DRN (up) and mPFC (down) slices. Scale bar 500 µm. **c)** Immunohistochemical successful colocalization of tdTom expression and α-SERT staining in mPFC slices. Scale bar 5 µm. **d)** Heatmaps of astrocyte ROIs, responding ROIs (n=56 out of 122, 45.9%, n=2 mice), and average population activity evoked by light stimulation of DRN afferences (10 s; 640-660 nm; red bar). Color code denotes fluorescence change. Data are presented as mean ± s.e.m. **e)** Box and whisker (BW) plots representing the dynamics of Ca^{2+} astrocytic events in response to 5-HT endogenous released. Data are shown as median ± range (min and max values). **f)** *Left,* representative EPSC average traces recorded from principal neurons in layer 2/3 mPFC in control mice before a), and after (b,c) puff application (black) and light stimulation (green). *Right*, average of normalized EPSC amplitude over time before and after local 5-HT puff (n=11 cells, n=7 mice) or endogenous release by DRN stimulation (n=7 cells, n=4 mice). **g)** Scatter plot of EPSC amplitude changes recorded during the first 5 min after puff/light stimulation, for short, and after 25 min for delayed synaptic effects. Note the similar transient synaptic depression of EPSCs amplitude induced by 5-HT puff application or 5-HT endogenous release. Paired-t-test, *P<0.05, **P<0.01, ***P<0.001.

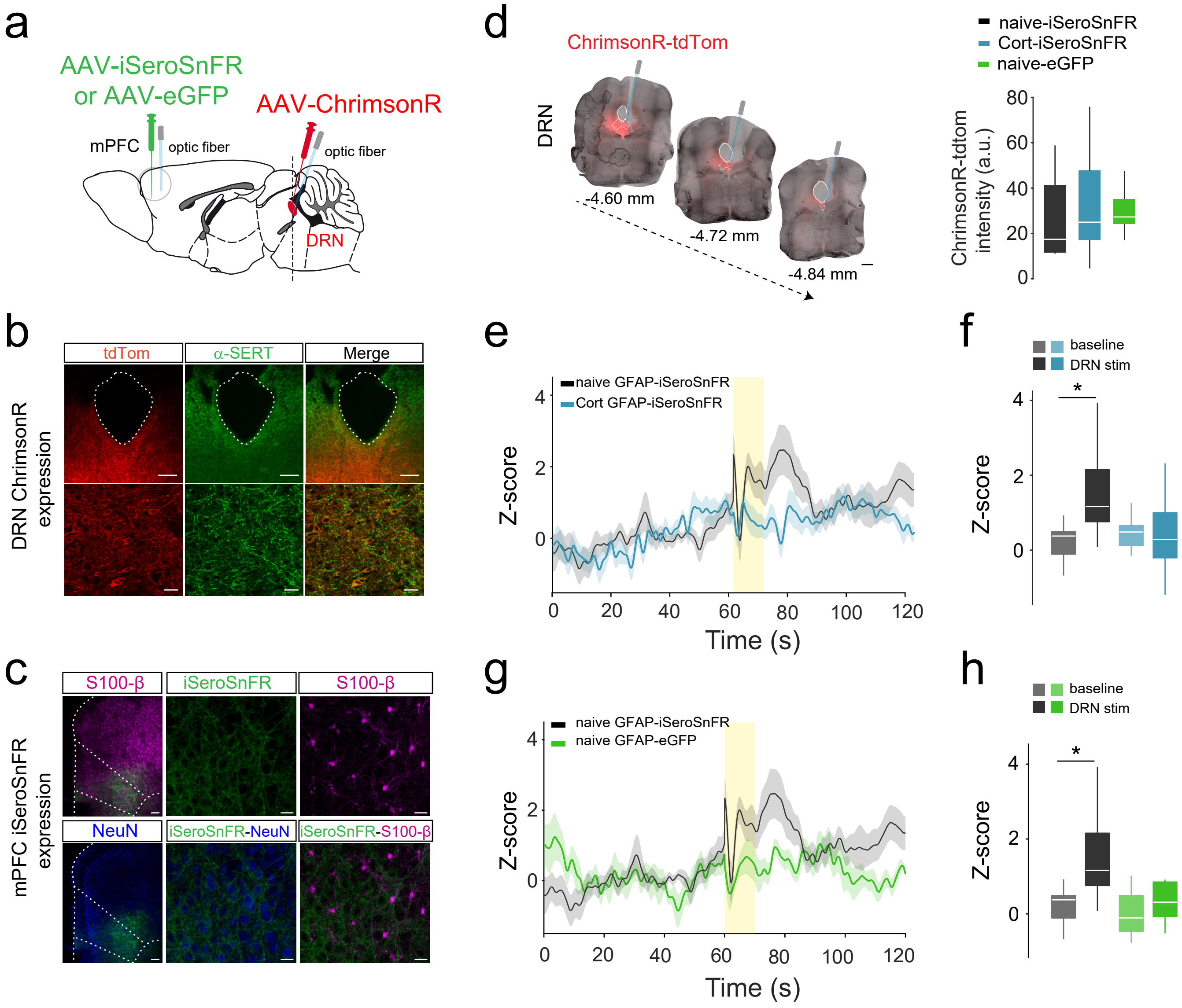

Extended Data Figure 5. Disrupted 5-HT-sensing by mPFC astrocytes in Cort-mice. a) Schematic representation of viral injections in mPFC area targeted with AAV5-GFAP-mcherry-Cre, AAV5-CAG-flex-iSeroSnFR and AAV5-GFAP-eGFP, and in DRN targeted with AAV9-hSyn-ChrimsonR-tdTom, showing the following optical fiber implantations. **b)** Confocal images showing AAV9-hSyn-ChrimsonR-tdTom (red) and α-SERT (green) colocalization in DRN. Scale bar, 100 µm (up), 20 µm (down). **c)** Immunohistochemical colocalization of iSeroSnFR (green), and S-100β (purple) staining in mPFC slices. Scale bar, 100 µm (left), 20 µm (right). **d)** *Left*. Representative coronal brain sections showing AAV9-hSyn-ChrimsonR-tdTom expression in DRN rostro-caudal axis. Scale bar, 500 µm. *Right*. Quantification of ChrimsonR-tdTom fluorescence intensity in DRN naïve-iSeroSnFR (black), Cort-iSeroSnFR (blue) and naïve-eGFP (green) mice. **e,g**) Average traces of fiber-photometry recordings in mPFC astrocytes in response to DRN light stimulation (yellow box, 10s at 40 Hz, 590 nm) in naïve-iSeroSnFR (gray), Cort-iSeroSnFR (blue), and naïve GFAP-eGFP mice (green). Data shown as mean ± s.e.m (shaded areas). **f, h**) Box and whisker (BW) plots representing fluorescence average 30 s before the stimulation onset (baseline) and 30 s after the beginning of the stimulation in naïve-iSeroSnFR (13 trials, n=3 mice), Cort-iSeroSnFR (20 trials, n=3 mice) and naïve-GFAP-eGFP mice (11 trials, n=3 mice). Note the increase in Ca^{2+} events amplitude in naïve-iSeroSnFR after DRN stimulation (P= 0.010), which was absent in Cort-iSeroSnFR and naïve-GFAP-eGFP mice (P=0,758; P= 0,234 respectively). One Way ANOVA, Holm-Sidak method, *P < 0.05. The center line in BW plots indicates the median, the top and bottom edges indicate the 25th and 75th percentiles, respectively, and the whiskers extend to the maximum and minimum data points.

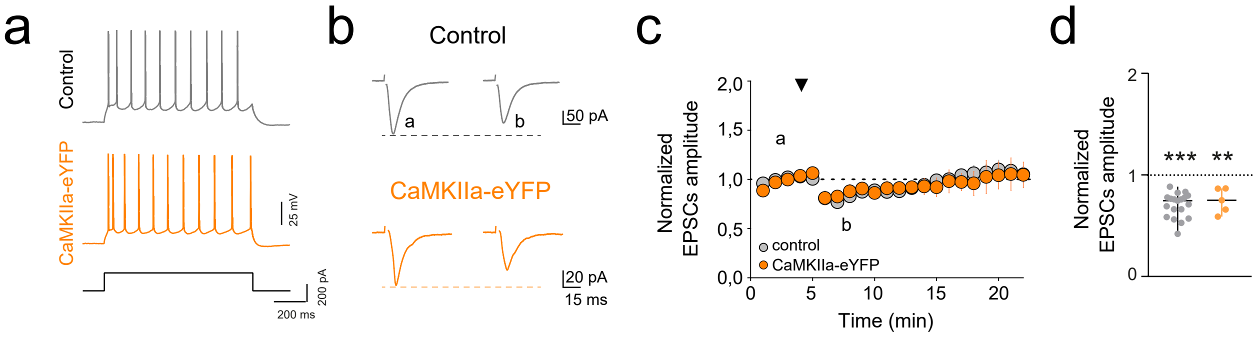

Extended Data Figure 6. CaMKIIa-eYFP expressing cells in layer 2/3 mPFC show transient synaptic depression of EPSCs. a) Representative action potential firing pattern of layer 2/3 principal cells from blind recordings (control, gray) and CaMKIIa-eYFP expressing neurons (orange) transfected with AAV5-CaMKIIa-eYFP virus. **b)** Representative EPSC average traces (average from 20 consecutive responses) before (a) and after (b) 5-HT application. **c)** Normalized EPSC amplitude over time before and after 5-HT stimulus in control (n=19 cells, n= 8 mice) and CaMKII-eYFP recording principal cells (n=5 cells, n= 2 mice). Black triangle denotes 5-HT local puff application (1 mM, 10 s, 1 bar). Data shown as mean ± s.e.m. **d)** Scatter plot of EPSC amplitude changes analyzed during the first 5 min after 5-HT puff application. Note the similar transient synaptic depression of EPSCs in both conditions. Paired-t-test, **P <0.01. ***P <0.001. Data are shown as median ± range (min and max values).

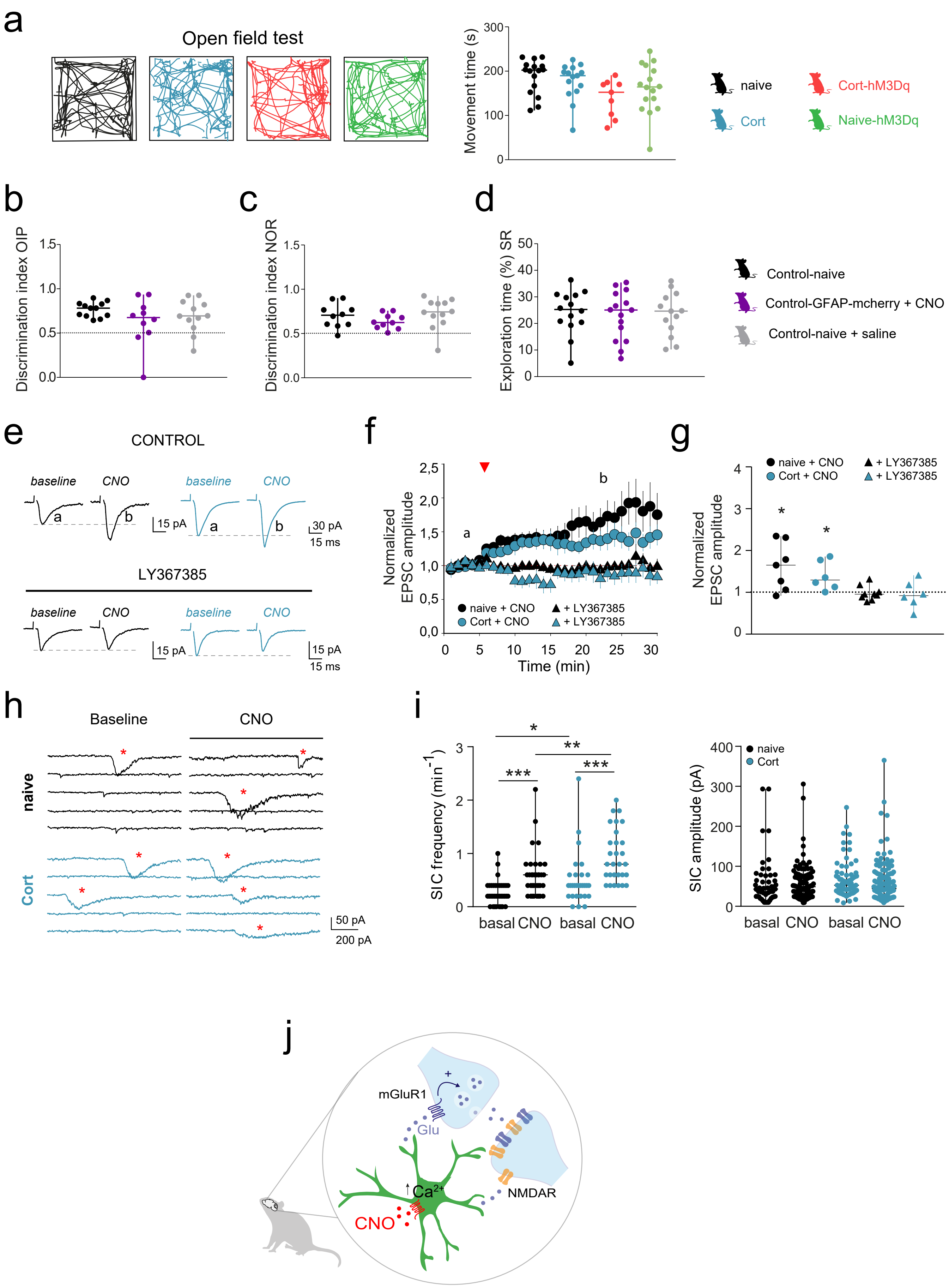
a
Open field test
Movement time (s)
naive
Cort
Cort-hM3Dq
Naive-hM3Dq
b
Discrimination index OIP
c
Discrimination index NOR
d
Exploration time (%) SR
Control-naive
Control-GFAP-mcherry + CNO
Control-naive + saline
e
CONTROL
baseline
CNO
15 pA
30 pA
15 ms
LY367385
f
Normalized EPSC amplitude
naive + CNO
Cort + CNO
+ LY367385
Time (min)
g
Normalized EPSC amplitude
h
Baseline
CNO
naive
Cort
50 pA
200 pA
i
SIC frequency (min^{-1})
basal CNO basal CNO
SIC amplitude (pA)
j
mGluR1
Glu
NMDAR
Ca^{2+}
CNO

Extended Data Figure 7. Gq-DREADDs activated astrocytes do not affect locomotion but stimulates cortical synaptic plasticity and gliotransmission. a) Animal tracking in OF arena in naïve, Cort-mice, Cort-GFAP-hM3Dq and naïve-GFAP-hM3Dq mice; and quantification OF total movement time. Note that mice mobility was not significantly affected among the experimental groups. One Way ANOVA, all pairwise, Dunn's Method, P> 0,05. Data shown median ± range (min and max values). **b,c,d)** OIP, NOR discrimination index and SR exploration time in naïve, vector transfected astrocytes mice (control-GFAP-mcherry) injected with CNO and naïve mice injected with saline. Note that cognitive performance and social interaction was preserved in control-GFAP-mcherry mice after CNO i.p. injection (3mg/kg, 20-30 min before behavioral testing) similar to control mice. One Way ANOVA, P>0.05. Data are shown as median ± range (min and max values). **e)** Representative EPSC average traces (average from 20 consecutive responses) recorded from principal neurons before (a), and after CNO application (b) in control and in the presence of LY367385 (100 μM) in naïve (black) and Cort-mice (blue). **f)** Normalized EPSC amplitude over time before and after hM3Dq activation of astrocytes in naïve (n=9 cells, n=6 mice) and Cort-mice (n=7 cells, n=3 mice), and naïve+LY367385 (n=12 cells, n=4 mice) and Cort-mice+LY367385 (n=11 cells, n=3 mice). Red triangle denotes CNO puff application (1 mM, 2s, 1 bar). Data shown as mean ± s.e.m. **g)** Scatter plot of EPSC amplitude changes recorded after 25 min of CNO puff. Note that hM3Dq astrocytic activation induced long-lasting enhancement of EPSCs in naïve and Cort-mice (Paired t-test, P= 0.028, P= 0.039 respectively), which was absent in presence of LY367385 (Paired t-test, P= 0.752, P= 0.694 respectively). *P<0.05. Data shown as median ± range. **h)** Representative traces of slow inward currents (SICs) before and after local CNO application (1 mM, 2s, 1 bar) in naïve and Cort-mice transfected with GFAP-hM3Dq DREADDS. Red asterisk indicates the presence of SICs. **i)** Scatter plot of SICs recorded before (basal, 5 min) and after CNO (5 min) showing a significant increase in frequency after CNO stimulation for both naïve (n=35 cells, n= 4 mice; One Way ANOVA, Tukey Test, P<0.001) and Cort-mice (n=29 cells, n= 3 mice; One Way ANOVA Tukey Test, P<0.001) with no effects in amplitude for both naïve and Cort-mice (One Way ANOVA, P>0.05). Note the enhanced SICs frequency in resting conditions (One Way ANOVA, Dunn's Method, P=0.013), and after CNO stimulation for Cort-mice vs naïve mice (One Way ANOVA, Dunn's Method, P=0.002). **j)** Schematic representation of CNO-driven molecular signalling in astrocytes and synaptic impact on excitatory synapses. Glutamate (Glu), mGluR1 receptors (blue), NMDAR (orange) and AMPAR (blue).

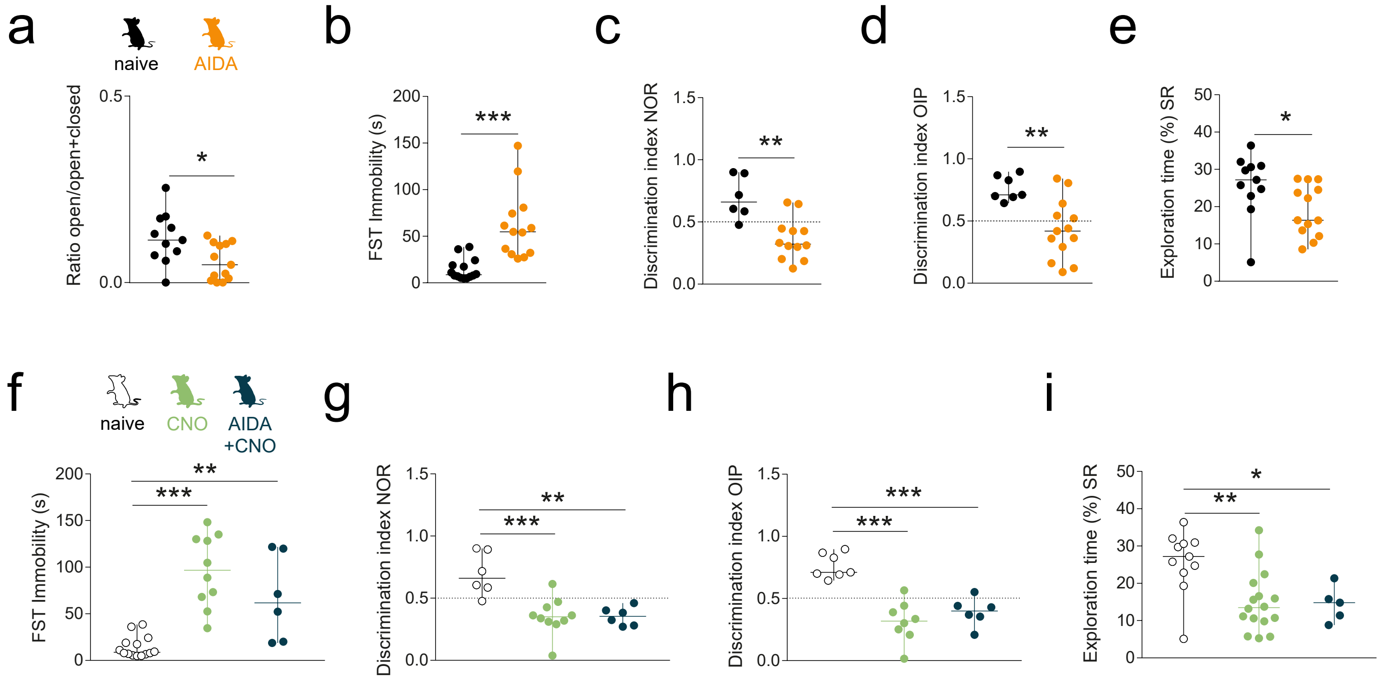

Extended Data Figure 8. mGluRI blockage impairs behavioral performance in naïve mice. a) EPM exploration index was reduced in naïve mice i.p. injected with AIDA, a selective metabotropic glutamate receptor 1 (mGluR1) antagonist (5 mg/kg; orange). One Way ANOVA, Tukey Test, P=0.014. **b)** Increased FST immobility time after AIDA administration compared to naïve mice, One Way ANOVA, Dunn's Method, P<0.001. **c,d,e)** NOR discrimination index, OIP discrimination index and SR exploration time were reduced after mGluRI blockage. One Way ANOVA, Tukey Test, P=0.001, P=0.003 and P=0.034, respectively. **f)** FST immobility time was increased after CNO i.p. administration (3 mg/kg; P=<0.001) in naïve mice (green), and in naïve mice previously treated with AIDA i.p. (P=0.004; dark blue). One Way ANOVA, Dunn's Method. **g,h,i)** NOR discrimination index, OIP discrimination index and SR exploration time were reduced after CNO and AIDA+CNO administration. One Way ANOVA, Holm-Sidak method, P<0.05. Data are shown as median ± range (min and max values). * P<0.05, **P <0.01, ***P <0.001.
